# Discovery of Benzophenanthridine Alkaloids from *Zanthoxylum nitidum* That Target the MDM2–p53 Axis in NSCLC

**DOI:** 10.3390/ph19060814

**Published:** 2026-05-22

**Authors:** Nguyen Manh Cuong, Elizaveta Fefilova, Vu Thanh Loc, Natalia Karpova, Nguyen Xuan Ha, Alexandra Daks, Nguyen Viet Ha, Tran Thu Huong, Sergey Parfenyev, Alexander Nazarov, Oleg Semenov, Yulia Gnennaya, Olga Fedorova, Nickolai A. Barlev, Oleg Shuvalov

**Affiliations:** 1 Institute of Chemistry, Vietnam Academy of Science and Technology, 18 Hoang Quoc Viet, Nghia Do, Hanoi 11353, Vietnam; 2 Institute of Cytology of the Russian Academy of Sciences, St. Petersburg 194064, Russia; e.fefilova@list.ru (E.F.);; 3 Faculty of Education, Hanoi Metropolitan University, 98 Duong Quang Ham, Nghia Do, Hanoi 11307, Vietnam; 4 Graduate University of Science and Technology (GUST), Vietnam Academy of Science and Technology, 18 Hoang Quoc Viet, Nghia Do, Hanoi 11353, Vietnam; 5 Department of Biomedical Sciences, School of Medicine, Nazarbayev University, Astana 010000, Kazakhstan; nikolai.barlev@nu.edu.kz; 6 National Laboratory of Astana, Astana 010000, Kazakhstan

**Keywords:** NSCLC, p53, MDM2, snail, *Zanthoxylum nitidum* (Roxb.) DC., nitidine, terihanine, sanguinarine, 8-acetonyldihydrochelerythrine, metabolic rewiring

## Abstract

**Background/Objectives:** Non-small cell lung cancer (NSCLC) accounts for about 85% of lung cancers and is a leading cause of cancer-related deaths worldwide. Pharmacological targeting of the p53–MDM2 interaction to activate wild-type p53 is a promising strategy for treating NSCLC that retain functional p53 (approximately 50% of all cases). **Methods:** We screened 33 ethnomedicinal Vietnamese plant extracts for their anticancer effects using p53-expressing and p53-null NSCLC cell models, as well as two non-cancerous cell lines for control. We used an array of different experimental approaches including NMR spectroscopy; molecular docking; an MTT test; cell cycle analysis; apoptosis analysis; wound healing, migration, and invasion assays; Real-Time PCR; immunoblotting; and Seahorse energy profiling to characterize and study the effects of these bioactive compounds on NSCLC cells. **Results:** Ethanol extract of *Zanthoxylum nitidum* stems and twigs demonstrated potent and selective activity by inducing p53-dependent cell cycle arrest and apoptosis. Phytochemical analysis identified several benzophenanthridine alkaloids as active constituents. Molecular docking revealed their strong in silico binding to MDM2. Notably, nitidine was the most promising compound among the molecules tested. Unlike nutlin, but similar to SP141 (two well-known MDM2 inhibitors), nitidine strongly stabilized p53 while concomitantly attenuating MDM2 at the protein level. Surprisingly, this effect was p53-independent. Additionally, nitidine suppressed the EMT master regulator Snail, and hence disrupted cellular bioenergetics and inhibited migration and invasion of NSCLC cells. **Conclusions:** Our findings identify *Z. nitidum* and nitidine as promising sources for developing novel MDM2-targeting therapeutics against NSCLC irrespective of the p53 status.

## 1. Introduction

Lung cancer remains one of the leading causes of cancer-related mortality worldwide, with non-small cell lung cancer (NSCLC) accounting for approximately 85% of all cases [1,2].

The treatment of non-small-cell lung cancer (NSCLC) has shifted from chemotherapy to precision medicine, driven by molecular discoveries like EGFR mutations, which led to tyrosine kinase inhibitors such as gefitinib and erlotinib [3]. Genomic profiling efforts, including The Cancer Genome Atlas (TCGA), have further defined the landscape of lung adenocarcinoma and squamous cell carcinoma. These efforts have enabled therapies for alterations in ALK, ROS1, and RET [4,5,6]. Immune checkpoint inhibitors like nivolumab and pembrolizumab have demonstrated superior efficacy, especially in tumors with high PD-L1 expression [7]. The evolution continues with third-generation agents, such as osimertinib for resistant EGFR mutations and alectinib for ALK-positive malignancies, which improve survival and quality of life [8]. This progression underscores the transformative impact of targeted immunotherapies guided by molecular oncology [9].

Despite advances in diagnosis and treatment, the prognosis for patients with non-small cell lung cancer (NSCLC) remains poor due to late-stage detection, high rates of recurrence, and resistance to conventional chemotherapy and targeted therapies. Therefore, identifying novel therapeutic targets and developing new agents is of great importance.

As a master tumor suppressor, p53 is a promising therapeutic target. In response to various forms of stress, the p53 protein undergoes multiple post-translational modifications [10,11,12] to activate transcription of non-coding [13] and protein-coding genes, including MDM2 [14]. Importantly, a significant proportion of NSCLC cases (35–77%) retain wild-type p53 (p53WT) [15,16]. In these malignancies, the tumor suppressor activity of p53 is primarily neutralized by its negative regulator, the E3 ubiquitin ligase MDM2. MDM2 binds to p53, promoting its ubiquitinylation and subsequent degradation in 26S proteasomes which, in turn, are also subjected to post-translational modifications [17]. Inhibiting the MDM2–p53 interaction can rapidly reactivate the potent p53 pathway, resulting in cell cycle arrest and apoptosis [18].

Beyond p53, MDM2 possesses a plethora of p53-independent oncogenic functions. It stabilizes a variety of oncogenes and degrades oncosuppressors [19,20,21]. Numerous experiments have shown that genetic or pharmacological MDM2 targeting usually induces the death of malignant cells and suppresses their migratory and invasive capabilities, as well as their stem cell-like features. This treatment also reverses their susceptibility to anticancer therapeutics [22,23,24]. Thus, a number of studies, including those published by our team [23,25], continue in the search to discover and develop new compounds and approaches targeting MDM2 to enhance the efficiency of anticancer therapy [26,27,28].

Medicinal plants have long been recognized as source of lead compounds for the discovery of new anticancer therapeutics [29,30,31,32,33,34]. These plants produce a vast array of structurally diverse secondary metabolites, including alkaloids, terpenoids, and polyphenols, with potent biological activities. Many chemotherapeutic agents used in clinical practice, such as vincristine, paclitaxel (Taxol), and camptothecin derivatives, were originally isolated from plants [35].

Vietnam’s tropical and subtropical ecosystems, ranging from the northern mountains to the Mekong Delta, harbor exceptional biodiversity. These ecosystems are home to thousands of plant species that are used in traditional medicine, known as Thuoc Nam [36,37]. The combination of ethnobotanical knowledge and chemical novelty makes these ecosystems a promising frontier for anticancer research, though systematic scientific validation is still in its early stages.

In this study, we screened 33 Vietnamese plant extracts used in traditional medicine for their anticancer activity using p53-null and wild-type p53 NSCLC cell models. Based on the obtained data, we further investigated the anticancer activities of stem and twig extracts of *Zanthoxylum nitidum* (Roxb.) DC. and their bioactive compounds, particularly their ability to modulate MDM2, p53, and Snail in NSCLC.

## 2. Results

### 2.1. Screening Vietnamese Ethnomedicinal Plant Extracts for Anticancer Activity in Non-Small Cell Lung Cancer (NSCLC) Cell Lines

We screened 33 extracts from 31 Vietnamese ethnomedicinal plants for their cytostatic and cytotoxic properties in three NSCLC cell lines (p53-null H1299, wild-type p53 A549, and H460) using an MTT assay. We used a wide range of concentrations, from 1 to 300 μg/mL. The detailed results are given in Table S1. Five of the 33 plant extracts were considered to have significant anticancer properties, including *Garcinia xanthochymus* Hook.f.ex T.Anderson leaves, *Zanthoxylum nitidum* (Roxb.) DC. stems and twigs, *Paramignya trimera* (Oliv.) Burkill roots, *Sarcandra glabra* (Thunb.) Nakai leaves, and *Pottsia laxifora* Kuntze stems. The remaining extracts showed either weak or no cytostatic/cytotoxic effects across all tested concentrations. However, these non-active samples nevertheless contribute important baseline data for future comparative studies and help to refine the pool of Vietnamese ethnomedicinal plants with potential anticancer relevance.

To assess the general toxicity and selectivity of these extracts, we screened them in two non-cancerous cell lines: human mesenchymal stem cells (DF2 and FRSN). According to this study, two extracts derived from *Garcinia xanthochymus* and *Zanthoxylum nitidum* demonstrated good selectivity, with selectivity index (SI) ranging from 2.5 to 10 for *Z. nitidum*. Additionally, extracts from *Paramignya trimera*, *Sarcandra glabra* and *Pottsia laxifora* demonstrated modest selectivity (Table S1).

**Figure 1:**
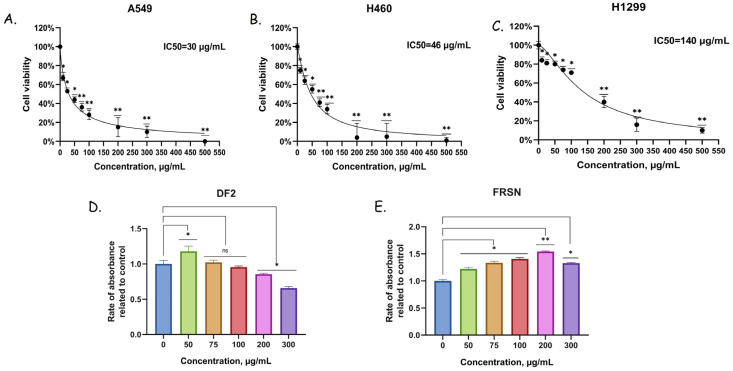
*Z. nitidum* ethanol extract selectively affects NSCLC cell lines. Results of MTT assay are shown. NSCLC cell lines. (**A**) A549 ells (p53+). (**B**) H460 cells (p53+). (**C**) H1299 cells (p53-null). Non-cancerous cell lines (mesenchymal stem cells). (**D**) DF2 cells. (**E**) FRSN cells. A Mann–Whitney U-test was applied to check for statistically significant differences. * *p* ≤ 0.05; ** *p* ≤ 0.01; ns—non-significant.

Based on the results of both efficiency and selectivity (Figure 1), we chose the ethanol extract of *Z. nitidum* stems and twigs for further study. As shown in Figure 1A,B, the ethanol extract of *Z. nitidum* suppressed p53+ A549 and H460 cell lines (IC_50_ = 30 and 46 μg/mL, respectively) three to five times more efficiently than p53- H1299 cells (IC_50_ = 140 μg/mL). Additionally, the same concentrations of the *Z. nitidum* ethanol extract did not affect non-cancerous cell lines (Figure 1D–E).

### 2.2. The Ethanol Extract of Z. nitidum Stabilizes p53 and Exhibits Multiple Anticancer Properties in NSCLC Cell Lines

**Figure 2:**
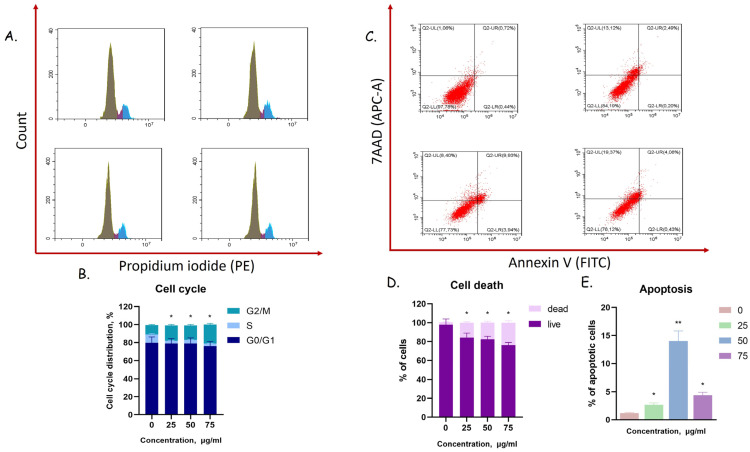
*Z. nitidum* ethanol extract induces cell cycle arrest (**A**,**B**) and apoptosis (**C**–**E**) in NSCLC A549 cell line upon 24 h treatment. Flow cytometry plots and quantitative diagrams are shown. Student’s *t*-test was applied to check pairwise statistically significant differences. * *p* ≤ 0.05; ** *p* ≤ 0.01.

We determined that the *Z. nitidum* extract was the most potent and selective for suppressing NSCLC lines. Next, we studied its impact on the cell cycle, cell death, and energy metabolism of cancer cells. As shown in Figure 2A,B, *Z. nitidum* caused cell cycle arrest by decreasing the percentage of cells in the S phase from 9% to 3% and increasing the percentage of cells in the G2/M phase from 10.5% to 21%. Furthermore, *Z. nitidum* induced cell death, increasing both necrosis (from 1.7% to 23%) and apoptosis (from 1.2% to 14%) (Figure 2C–E).

Several constituents of *Z. nitidum* have been shown to activate the p53 signaling pathway [38,39]. Therefore, we studied the impact of its extract on the MDM2–p53 axis. MDM2, an E3 ubiquitin ligase, is the main negative regulator of p53, targeting it for constitutive proteasomal degradation [18]. Stabilization and increased levels of the p53 protein are usually associated with disruption of the MDM2–p53 interaction, which can result from post-translational modifications of p53 and MDM2 or pharmacological inhibition of their binding [25].

**Figure 3:**
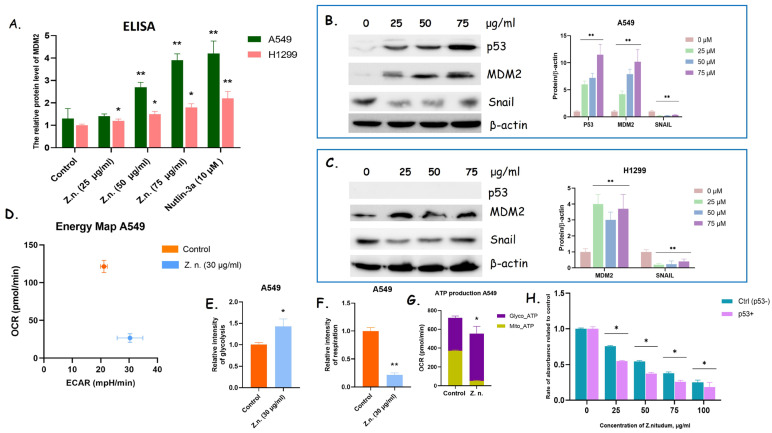
*Z. nitidum* ethanol extract affects p53–MDM2 axis and bioenergetics in NSCLC cell lines. (**A**) ELISA test demonstrating *Z. nitidum* extract increases MDM2 protein level in A549 and H1299 cells. Z. n.—*Zanthoxylum nitidum*. Nutlin-3a—a well-known inhibitor of the p53–MDM2 interaction. Immunoblots showing the impact of *Z. nitidum* extracts on p53, MDM2, and Snail in (**B**) A549 and (**C**) H1299 cell lines. The diagrams show the results of quantification performed using the Image J software (version 1.54r). Student’s *t*-test was used to determine pairwise statistically significant differences. Asterisks represent a significant difference in protein expression (* *p* ≤ 0.05; ** *p* ≤ 0.01). Results of SeaHorse profiling demonstrating (**D**) the energy map, the impact of *Z. nitidum* extract on (**E**) the intensity of glycolysis and (**F**) respiration relative to control (DMSO-treated) cells. (**G**) The rate of ATP production by glycolysis (Glyco_ATP) and oxidative phosphorylation (Mito_ATP). (**H**) Results of MTT assay, showing the increased susceptibility of H1299 cells to *Z. nitidum* extract upon tetracyclin-induced (TetOn) expression of exogeneous wild-type p53. A Mann–Whitney U-test was applied to check for statistically significant differences. * *p* ≤ 0.05; ** *p* ≤ 0.01.

First, we performed an ELISA assay to quantify the level of MDM2 protein after treating cells with *Z. nitidum* extract or nutlin-3a, the standard inhibitor of the MDM2–p53 interaction. As shown in Figure 3A, the *Z. nitidum* extract and nutlin-3a had comparable effects, inducing a 1.2- to 4-fold increase in MDM2 protein levels, particularly in p53-positive A549 cells. Additionally, we performed a Western blot analysis of MDM2 and p53. As Figure 3B,C demonstrate, the *Z. nitidum* extract significantly increased the levels of both MDM2 and p53 proteins, which aligns with the ELISA data.

Previous research has demonstrated that MDM2 can induce Snail, a key transcription factor that promotes epithelial-to-mesenchymal transition (EMT) in cancer cells [40,41]. Furthermore, MDM2 inhibitors decrease Snail protein levels and suppress EMT [42,43,44,45]. We checked the impact of MDM2 on Snail in A549 cells and demonstrated that MDM2 knockdown significantly suppresses Snail protein levels (Figure S1). Therefore, we evaluated the effect of the *Z. nitidum* extract on Snail using Western blot analysis. Figure 3C shows a 2–3 fold reduction in Snail protein levels in both the A549 and H1299 cell lines, suggesting a negative impact on EMT and migration properties.

We recently demonstrated that MDM2 upregulates energy metabolism in non-small cell lung cancer (NSCLC) cell lines in a p53-independent manner, revealing its role in metabolic rewiring [23]. Since metabolic rewiring, including deregulated energy metabolism, is considered one of the “hallmarks of cancer” and a potential drug target [30,46], we carried out Seahorse profiling and analyzed the impact of *Z. nitidum* extract on glycolysis and respiration intensities. As can be seen in Figure 3D–F, the energy map and diagrams show that the *Z. nitidum* extract greatly affected respiration, suppressing it 4.5 times. At the same time, glycolysis increased 1.4-fold, which could compensate for the strong inhibition of respiration. Overall, this results in significant inhibition of ATP production (Figure 3G). However, the increase in glycolysis, even as a compensatory response to suppressed respiration, does not seem desirable for anticancer implementation.

*Z. nitidum* extract affected both the A549 (p53) and H1299 (p53-) cell lines. However, the results of the MTT test, cell cycle assay, apoptosis assay, and Seahorse profiling showed higher activity in the A549 (p53+) cell line (see Figure 1, Figure 2 and Figure 3). These results suggest the potential impact of intact p53. To test this theory, we performed an MTT assay using H1299 cells with TetOn-based, lentiviral-inserted, induced expression of exogenous wild-type p53 [47]. Treating the cells with doxycycline to induce p53 expression significantly increased the susceptibility of H1299 cells to the extract by 5–20%, depending on the concentration (Figure 3H). Thus, *Z. nitidum* induces p53 and MDM2 stabilization, and its toxicity increases in the presence of wild-type p53.

### 2.3. Identification of Compounds from the Stems and Twigs of Zanthoxylum nitidum

**Figure 4:**
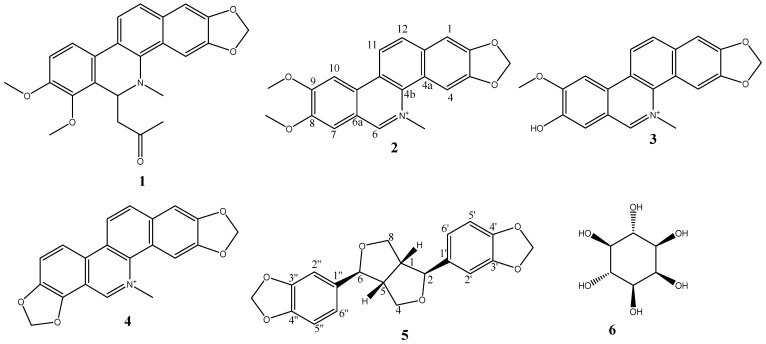
Isolated compounds from the stems and twigs of *Zanthoxylum nitidum*.

In order to identify bioactive compounds, repeated column chromatography of the 80% ethanol fraction of *Z. nitidum* stems resulted in the isolation of six known compounds (**1**–**6**), including three benzophenanthridine alkaloids: 8-acetonyldihydrochelerythrine (**1**), nitidine (**2**), terihanine (**3**), and sanguinarine (**4**) as well as a lignan: sesamin (**5**), and a cyclitol: myo-inositol (**6**) (Figure 4). Their chemical structures were elucidated based on NMR data, and comparisons with literature data. The observed HR-ESI-MS data for all compounds were consistent with their molecular formulas: *m*/*z* 406.16490 [M+H]^+^ (calcd. for C_24_H_24_NO_5_^+^ (**1**), 406.16543, Δ = −1.3 ppm) (Figures S2–S5); *m*/*z* 349.13086 [M+H]^+^ (calcd. for C_21_H_19_NO_4_^+^ (**2**), 349.13140, Δ = −1.5 ppm) (Figures S6–S8); *m*/*z* 335.11521 [M+H]^+^ (calcd. for C_20_H_17_NO_4_^+^ (**3**), 335.11570, Δ = −1.5 ppm) (Figures S9–S11); *m*/*z* 333.09956 [M+H]^+^ (calcd. for C_20_H_15_NO_4_^+^ (**4**), 333.10010, Δ = −1.6 ppm) (Figures S12–S15); *m*/*z* 353.13153 [M−H]^−^ (calcd. for C_20_H_17_O_6_^−^ (**5**), 353.10250, Δ = 82.2 ppm) (Figures S16 and S17); and *m*/*z* 179.05620 [M−H]^−^ (calcd. for C_6_H_11_O_6_^−^ (**6**), 179.05550, Δ = 3.9 ppm) (Figures S18 and S19). The HPLC chromatographic analysis indicated purities ≥ 97% for all tested compounds.

### 2.4. Molecular Docking of Isolated Compounds with MDM2

Upon observing *Z. nitidum*-mediated stabilization of both MDM2 and p53, we questioned its potential impact on the MDM2–p53 protein–protein interaction. Molecular docking results revealed that the benzophenanthridine alkaloids from *Z. nitidum* exhibited favorable binding affinities toward the MDM2 protein, ranging from −8.106 to −9.187 kcal/mol (Table 1).

**Figure 5:**
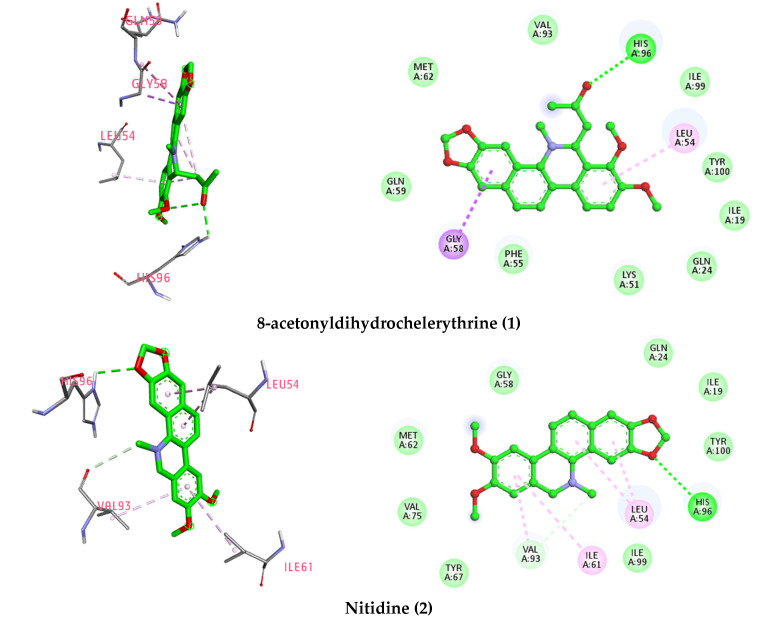

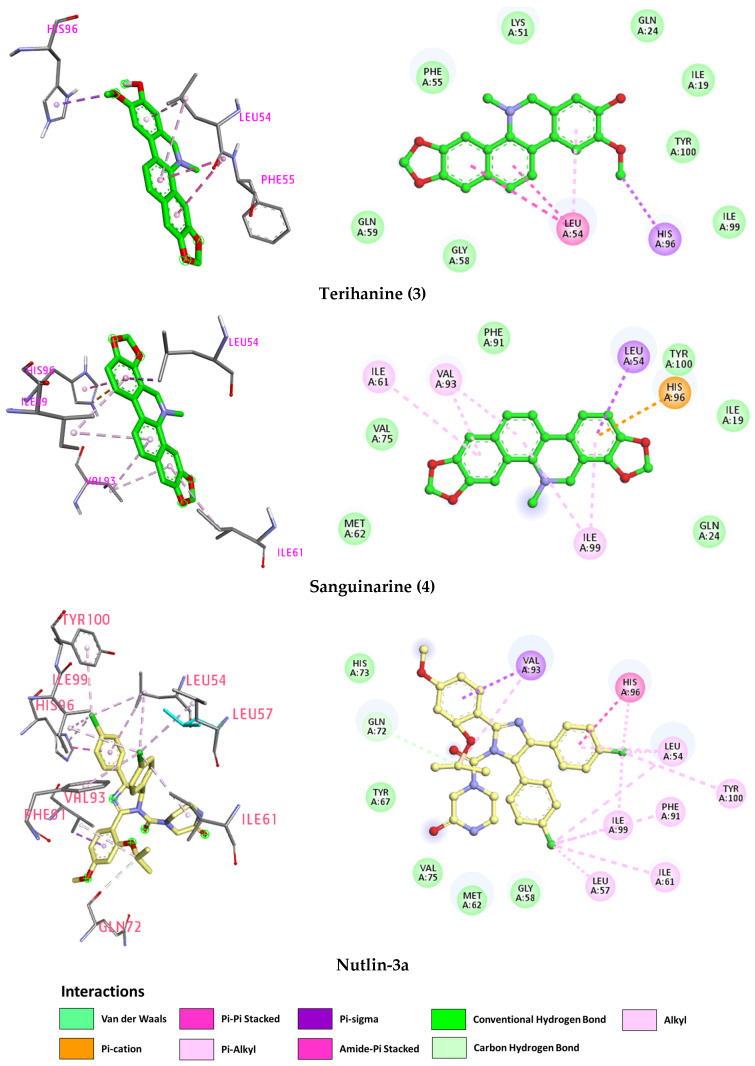
Binding interactions between studied benzophenanthridine alkaloids and the MDM2 protein (PDB ID: 4HG7), visualized using the Discovery Studio Visualizer software. The left panels show the 3D docking poses of the ligands within the MDM2 binding pocket, while the right panels illustrate the 2D interaction diagrams.

Among them, sanguinarine (**4**) showed the strongest binding affinity (−9.187 kcal/mol), suggesting a high potential to inhibit the ubiquitin ligase activity of MDM2. Its interactions included π–alkyl contacts with Ile61, Val93, and Ile99, a π–σ interaction with Leu54, and a notable π–cation interaction with His96, an amino acid located near the p53-binding cleft of MDM2 (Figure 5) [48]. These interactions could contribute to strong stabilization within the binding pocket, potentially interfering with the MDM2–p53 interaction. Nitidine (**2**) and 8-acetonyldihydrochelerythrine (**1**) also demonstrated substantial binding affinities (−8.106 and −8.304 kcal/mol, respectively). Nitidine formed a hydrogen bond with His96 in addition to π–alkyl contacts, while compound **1** established both hydrogen bonding and hydrophobic interactions (π–alkyl, π–σ) with key residues (Leu54, Gly58, His96), which are critical for p53 recognition (Figure 5). Terihanine (**3**) exhibited similar affinity (−8.491 kcal/mol) and engaged in amide–π stacked and π–alkyl interactions with Leu94 and His96, contributing to moderate stabilization within the pocket (Figure 5).

### 2.5. Benzophenanthridine Alkaloids from Zanthoxylum nitidum Affect the Levels of p53, MDM2, and Snail Proteins, as Well as Bioenergetics, in NSCLC Cell Lines

First, we examined the cytotoxic and cytostatic properties of the following compounds in NSCLC cell lines using an MTT assay: isolated benzophenanthridine alkaloids (nitidine, terihanine, and 8-acetonyldihydrochelerythrine) and the lignan sesamin. The results, shown in Table 2, demonstrate that sanguinarine was the most potent (IC_50_ ranges from 1.5 to 1.8 μM), followed by nitidine (IC_50_ 25–45 μM). The other compounds did not display significant inhibitory activity in NSCLC cell lines.

**Figure 6:**
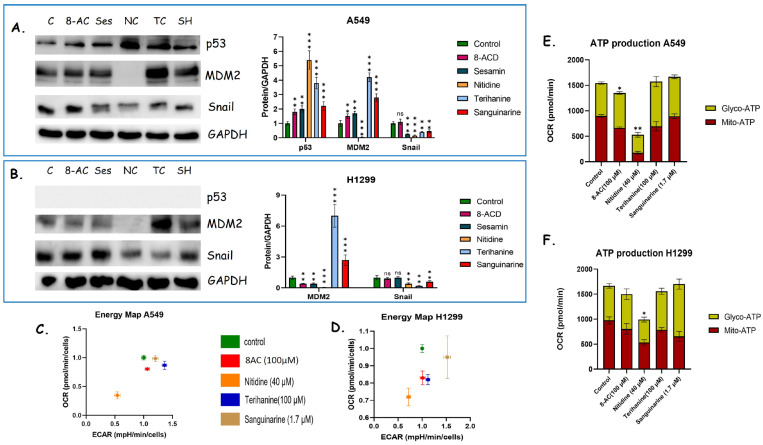
Benzophenanthridine alkaloids isolated from *Z. nitidum* affect p53, MDM2, Snail, glycolysis, and respiration in NSCLC cell lines. Immunoblots demonstrating the impact of 8-acetonyldihydrochelerythrine (8-AC), sesamin (Ses), nitidine (NC), terihanine (TC), and sanguinarine (SC) on (**A**) A549 and (**B**) H1299 cell lines. C—Control (DMSO-treated) cells. Cells were treated with indicated concentrations for 24 h. The diagrams show the results of quantification performed using the Image J software. Student’s *t*-test was used to determine pairwise statistically significant differences. Asterisks represent a significant difference in protein expression (* *p* ≤ 0.05; ** *p* ≤ 0.01; *** *p* ≤ 0.001). Results of SeaHorse profiling demonstrating the impact of 24 h treatment of NSCLC cell lines with benzophenanthridine alkaloids. The energy diagrams of (**C**) A549 and (**D**) H1299 cells. OCR—Oxygen Consumption Rate (indicates respiration); ECAR—Extracellular Acidification Rate (indicates glycolysis). The diagrams showing ATP production rate in (**E**) A549 and (**F**) H1299 cell lines. A Mann–Whitney U-test was applied to check for statistically significant differences. * *p* ≤ 0.05; ** *p* ≤ 0.01; *** *p* ≤ 0.001; ns—nonsignificant.

Having clearly linked *Z. nitidum* extract to the p53–MDM2 protein–protein interaction, we next assessed the impact of compounds **1–5** on the levels of MDM2, p53, and Snail proteins. As shown in Figure 6A,B, all compounds stabilized p53 to varying degrees; however, nitidine and terihanine were the most effective. Regarding MDM2, however, these two compounds had the opposite effect. While terihanine stabilized MDM2 like its well-known inhibitor nutlin-3a, nitidine, on the other hand, dramatically down-regulated MDM2’s protein level (Figure 6A,B), similar to another well-known inhibitor, SP141 [22]. Furthermore, nitidine and terihanine both decreased Snail protein levels by three to four times (Figure 6A,B).

Then, we studied the impact of compounds **1–4** on the energy metabolism of the A549 (p53+) and H1299 (p53-) NSCLC) cell lines using Seahorse energy profiling. Interestingly, all compounds except sanguinarine mitigated respiration in both cell lines; however, nitidine demonstrated the best inhibitory activity, strongly suppressing both glycolysis and respiration (Figure 6C,D). In fact, nitidine was the only compound that significantly reduced both glycolytic and mitochondrial ATP production by three times, indicating a strong inhibitory impact on bioenergetics and metabolic rewiring in NSCLC cells (Figure 6E,F).

Based on the results, we chose nitidine as the most promising compound for further study.

### 2.6. Nitidine from Z. nitidum Affects p53/MDM2/Snail/β-Catenin Axis in NSCLC Cell Lines

We decided to investigate the molecular mechanisms by which nitidine impacts the p53/MDM2/Snail axis in greater detail.

First, to verify that nitidine can physically bind to MDM2, we performed a co-immunoprecipitation of HA-tagged MDM2 with endogenous p53 in DMSO- and nitidine-treated A549 cells. Nutlin-3a was used as control. Expectedly, nutlin-3a disrupted the MDM2–p53 interaction, resulting in a 5-fold decrease. Nitidine also reduced this interaction, albeit by only 2-fold. Although not as clear as in the case of nutlin-3a, these data demonstrate the ability of nitidine to disrupt the MDM2–p53 interaction (Figure S20).

**Figure 7:**
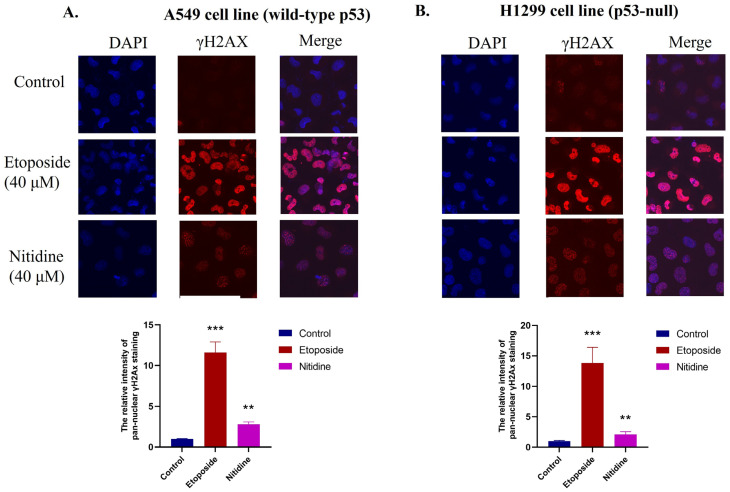
Nitidine induced slight DNA damage in comparison with etoposide. (**A**) A549 (wild-type p53) and (**B**) H1299 (p53-null) cells were treated with 40 μM of nitidine or etoposide for 4 h, followed by immunostaining for γH2AX (red) and confocal microscopy. DMSO was used as a control. Nuclei were stained with DAPI (blue). The intensity of pan-nuclear γH2AX staining was analyzed using the ImageJ software. Student’s *t*-test was used to determine pairwise statistically significant differences. Asterisks represent a significant difference in γH2AX intensity (** *p* ≤ 0.01; *** *p* ≤ 0.001).

Several studies have demonstrated that nitidine can induce genotoxic stress [38,49,50], which in turn also activates p53. To delineate the mechanism of nitidine-dependent activation of p53, we compared the ability of nitidine to promote DNA damage with that of a known genotoxic agent, etoposide, in p53+ versus p53-negative cells. To this end, we treated A549 cells, which express p53 wild-type and p53-null H1299 cells with the same concentrations (40 μM) of nitidine or etoposide, followed by γH2AX immunostaining and confocal microscopy. The results shown in Figure 7 demonstrate a very slight activation of DNA damage induced by nitidine compared to etoposide. Whereas etoposide increased the pan-nuclear γH2AX staining 11.6-fold in A549 cells and 13.8-fold in H1299 cells, respectively, nitidine induced only a 2.8-fold and 2.1-fold increase in these cell lines. These results suggest that nitidine is a much less potent inducer of DNA damage than etoposide and, hence, its effects on p53 are likely to be DNA damage-independent.

**Figure 8:**
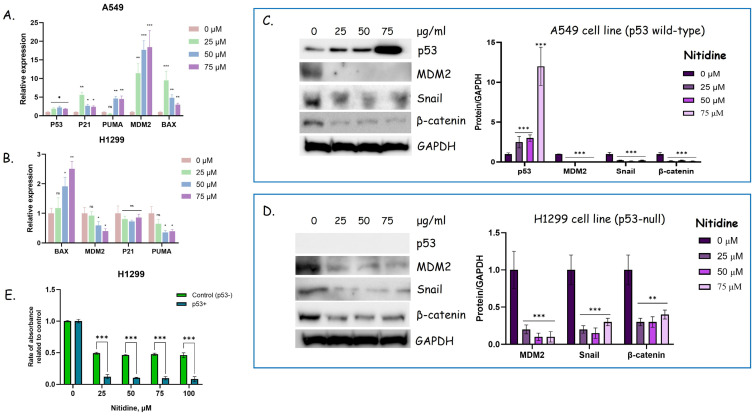
Nitidine from *Z. nitidum* affects p53/MDM2/Snail/β-catenin axis in NSCLC cell lines. Real-time PCR showing the impact of nitidine on mRNA level of *p53* and its transcription targets (MDM2, *p21*, *Bax*, *and Puma*) in (**A**) A549 and (**B**) H1299 cell lines. Immunoblots demonstrating the impact of nitidine treatment on protein levels of p53, MDM2, Snail, and β-catenin in (**C**) A549 and (**D**) H1299 cell lines. The diagrams show the results of quantification performed using the Image J software. Student’s *t*-test was used to determine pairwise statistically significant differences. Asterisks represent a significant difference in protein expression (* *p* ≤ 0.05; ** *p* ≤ 0.01; *** *p* ≤ 0.001). (**E**) Results of MTT assay, showing the increased susceptibility of H1299 cells to nitidine upon tetracyclin-induced (TetOn) expression of exogeneous wild-type p53. A Mann–Whitney U-test was applied to check for statistically significant differences. * *p* ≤ 0.05; ** *p* ≤ 0.01; *** *p* ≤ 0.001; ns—nonsignificant.

Next, we treated the wild-type A549 and p53-deficient H1299 NSCLC cell lines with increasing concentrations (25, 50, and 75 μM) of nitidine for 16 h. Then, we performed a real-time PCR assay to quantify the impact of nitidine on p53 and its well-known transcriptional targets: MDM2, p21, Bax, and Puma. As shown in Figure 8A,B, nitidine significantly increased the mRNA levels of the p53 targets MDM2, p21, Bax, and Puma in the wild-type p53 A549 cells, which is consistent with the data on p53 stabilization at the protein level. In p53-null H1299 cells, nitidine only upregulated Bax, while slightly inhibiting the expression of Puma and MDM2.

Next, we analyzed the impact of nitidine on the protein levels of p53, MDM2, and Snail. We also included in our analysis the H1975 cell line bearing mutant p53. As shown in Figure 8C,D, nitidine upregulated the expression of p53 at the protein level by four to twelve times only in p53-positive A549 cells. In contrast, nitidine drastically suppressed MDM2 levels both in A549 and p53-null H1299 cells. Although no significant impact on the protein level of mutant p53 was observed in H1975 cells, nitidine still decreased the level of MDM2 in this cell line (Figure S21). Consistent with our previous data (Figure S1), the protein level of Snail was also dramatically reduced in all three cell lines treated with nitidine (Figure 8C,D and Figure S21).

It has previously been shown that MDM2 downregulation is associated with increased β-catenin degradation [45]. *β*-catenin is the central effector of the canonical Wnt signaling pathway, which forms a positive feedback loop with Snail and cooperates with it to drive EMT, migration, and invasion [51]. In light of this, we analyzed nitidine’s impact on β-catenin at the protein level and demonstrated its significant downregulation in all cell lines tested (Figure 8C,D and Figure S21).

To study the requirements of wild-type p53 for nitidine activity, we used H1299 cells with doxycycline-inducible exogeneous p53 expression. Pretreating the cells with doxycycline dramatically increased their sensitivity to nitidine, suggesting that intact p53 signaling significantly determines the response rate to nitidine (Figure 8E).

**Figure 9:**
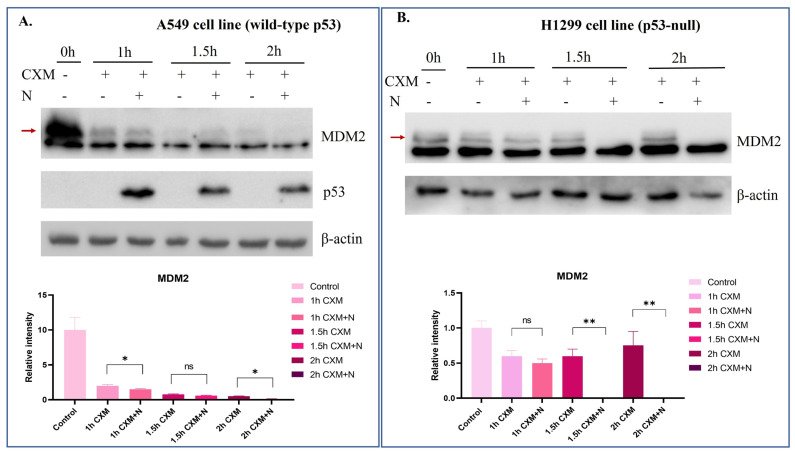
Nitidine destabilizes MDM2 protein. (**A**) A549 and (**B**) H1299 cells were treated with nitidine (N, 20 μM), and then exposed to cycloheximide (CHX, 50 μg/ml), a translation inhibitor, for the indicated time periods. The protein levels of MDM2 and p53 were assessed by Western blot. Arrows indicate true MDM2 band. The diagrams show the results of quantification performed using the ImageJ software. Student’s *t*-test was used to determine pairwise statistically significant differences. Asterisks represent a significant difference in protein expression (* *p* ≤ 0.05; ** *p* ≤ 0.01; ns—non-significant).

To further investigate the molecular mechanism underlying the negative impact of nitidine on MDM2 protein levels, we treated A549 and H1299 cells with nitidine for 24 h, followed by the addition of cycloheximide (CHX), an inhibitor of translation elongation. As shown in Figure 9, nitidine significantly increased MDM2 protein degradation in both cell lines, while also stabilizing p53 in the A549 cell line.

**Figure 10:**
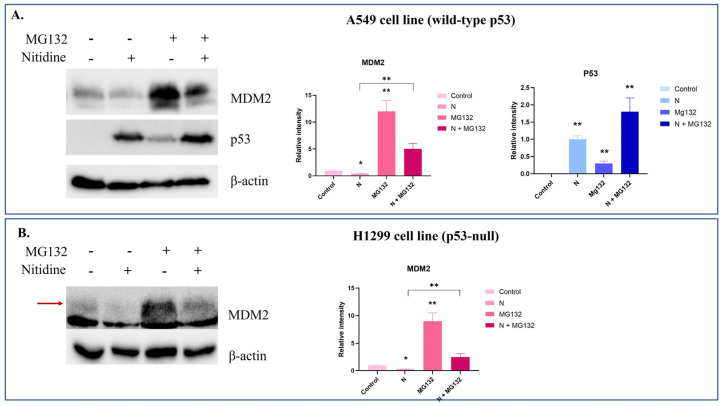
Proteasome inhibitor MG132 reverses nitidine-induced MDM2 degradation. (**A**) A549 cell line. (**B**) H1299 cell line. Cells were treated with nitidine (N, 25 μM) and MG132 (a proteasome inhibitor, 10 μM), either individually or in combination. The protein levels of MDM2 and p53 were assessed by Western blot. The arrow indicates true MDM2 band. The diagrams show the results of quantification performed using the ImageJ software. Student’s *t*-test was used to determine pairwise statistically significant differences. Asterisks represent a significant difference in protein expression (* *p* ≤ 0.05; ** *p* ≤ 0.01).

To investigate the involvement of proteasomes in nitidine-mediated MDM2 destabilization, H1299 and A549 cells were treated with nitidine and MG132 (a proteasome inhibitor), either individually or in combination (Figure 10). Nitidine alone decreased MDM2 protein level. However, co-treatment with nitidine and MG132 restored the MDM2 protein level, suggesting that proteasomes are involved in nitidine-induced MDM2 destabilization (Figure 10).

### 2.7. Nitidine Augments ROS Production and Autophagy While Suppressing Migration and Invasion

We have shown that nitidine dramatically supresses MDM2, Snail and β-catenin, as well as suppresses both glycolysis and OXPHOS, thus dramatically affecting ATP production. To obtain mechanistic insight into how metabolic collapse and down-regulation of oncogenes link to cell features, we assessed the impact of nitidine on ROS production, autophagy, migration and invasion capabilities.

**Figure 11:**
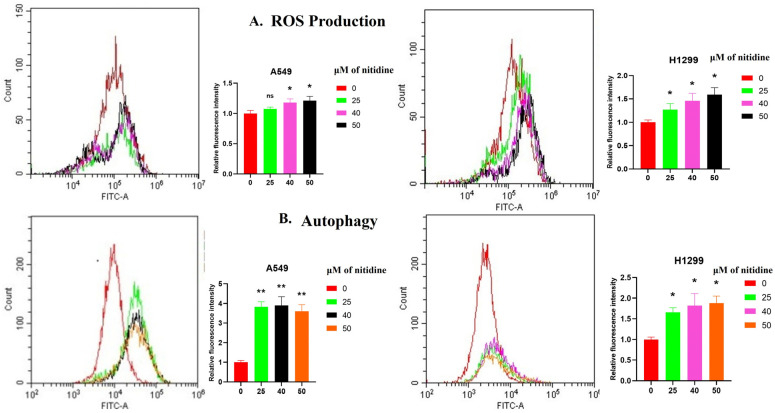
Nitidine increases ROS production and autophagy. Flow cytometry plots and quantitative diagrams are shown. A Student’s *t*-test was applied to check for statistically significant pairwise differences (* *p* ≤ 0.05; ** *p* ≤ 0.01; ns—non-significant).

Using flow cytometry, we revealed the nitidine-induced increase in ROS production to 20% and 60% in A549 and H1299 cell lines, respectively (Figure 11). Furthermore, nitidine dramatically increased autophagy in A549 cells (3.6–3.9 times) whereas its impact in H1299 cells was mode moderate (1.6–1.9 times).

**Figure 12:**
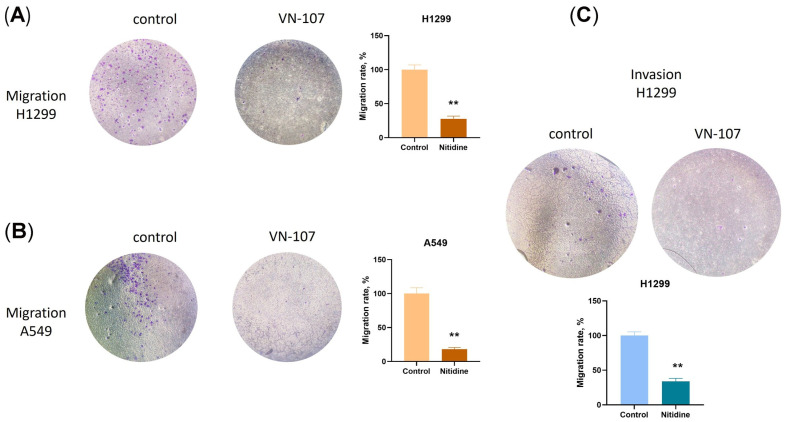
Nitidine dramatically suppresses the migratory and invasion capabilities of NSCLC cell lines. H1299 (p53-null) and A549 (wild-type 53) cells were treated with 40 μM nitidine or DMSO (control) for 24 h, followed by transwell migration assay (8 μm pores) and invasion assay (8 μm pores coated with Matrigel). The percentages of migrated and invaded cells were normalized to cell viability, which was assessed in parallel by trypan blue staining. Representative images and bar graphs illustrate the normalized migration assay results for the H1299 (**A**) and A549 (**B**) cell lines and the invasion assay results for the H1299 (**C**) cell line (** *p* ≤ 0.01).

We suggested that a dramatic decrease in Snail and β-catenin could affect the migration and invasion capabilities of cells. To test this hypothesis, we treated cells with 40 μM nitidine for 24 h and performed a wound-healing test, as well as transwell migration and invasion assays. The wound-healing assay showed a 91% and 75% reduction in motility for the A549 and H1299 cell lines, respectively (Figure S22). Consistent with these findings, the transwell migration assay revealed a 94% and 82% reduction in migration for these cell lines, respectively (Figure 12A,B). Finally, nitidine inhibited H1299 cell invasion capabilities by 78% (Figure 12C). Taken together, these data suggest that nitidine dramatically reduces the migratory and invasive potential of NSCLC cell lines.

## 3. Discussion

In the present study, we identified *Zanthoxylum nitidum*, a medicinal plant, as a promising source of novel anticancer agents. The principal constituents of the plant are benzophenanthridine alkaloids. Through systematic screening of Vietnamese medicinal plant extracts, we demonstrated that the ethanol extract of *Z. nitidum* stems and twigs exhibits potent, selective cytotoxicity against non-small cell lung cancer (NSCLC) cell models compared to non-cancerous mesenchymal stem cell lines.

Members of the genus *Zanthoxylum* (Rutaceae) are well recognized among medicinal plants for their diverse bioactivities, including anti-inflammatory, antimicrobial, and antitumor effects [52]. *Z. nitidum* is a scandent, prickly shrub distributed across the Molucca Islands, New Guinea, South China, and Vietnam. In traditional medicine, its roots are used to promote blood circulation, alleviate blood stasis, and treat snakebites. The leaves and stems are commonly used to treat toothaches, sore throats, coughs, and fevers. Phytochemical investigations have revealed that the plant is rich in alkaloids, lignans, and coumarins, which exhibit diverse pharmacological activities [53].

For example, the benzophenanthridine alkaloid 7-methoxy-8-demethoxynitidine exhibits potent cytotoxic activity against various human cancer cell lines [54]. Recently, Qin et al. isolated eleven previously undescribed isoquinoline alkaloids and twenty known compounds from the ethanolic extract of *Z. nitidum*. Among these compounds, (1′S,6R)-nitidumalkaloid B exhibited the most potent antiproliferative effects. It induced G_2_/M cell cycle arrest, apoptosis, and inhibition of the Wnt/*β*-catenin signaling pathway. It also suppressed cell migration by modulating epithelial–mesenchymal transition [55]. Together, these findings establish *Z. nitidum* as a promising source for developing new anticancer and antimicrobial agents.

In the present research, *Z. nitidum* extract induced G2/M cell cycle arrest, apoptosis, and profound suppression of mitochondrial respiration, coupled with an increase in compensatory glycolysis. At the molecular level, the extract significantly stabilized both p53 and MDM2, the main E3 ubiquitin ligase of p53 in A549 cells. Furthermore, the extract stabilized MDM2 in p53-null H1299 cells, suggesting potential inhibition of its self-ubiquitinylation capabilities.

Recently, we demonstrated that MDM2 mediates the upregulation of energy metabolism in NSCLC cells in a p53-independent manner [23]. Previously, other authors reported the important role of MDM2 in supporting serine metabolism [56,57]. Metabolic rewiring, including alterations in energy metabolism, one-carbon metabolism, glutaminolysis, de novo lipogenesis, and other metabolic processes and their regulation, is recognized as one of the hallmarks of cancer and a therapeutic target [30,46,47,58].

Consistent with this, we demonstrated a dramatic reduction in respiration upon treatment with *Z. nitidum* extract in a p53-independent manner. However, the increase in compensatory glycolysis diminishes the extract’s potential therapeutic value.

In addition, MDM2 is involved in regulating EMT. One proposed mechanism is its impact on Snail, the master regulator of the EMT program [59] which, in turn, promotes metastasis, cancer stem cell-like features, and resistance to therapy [60,61,62,63]. Although one study has shown MDM2-mediated polyubiquitination and degradation of Snail in hepatocellular carcinoma [64], several other studies have demonstrated MDM2’s positive impact on Snail protein levels and EMT features. These features include downregulation of E-cadherin and upregulation of vimentin and N-cadherin, as well as increased motility and invasion [40,41,65]. Furthermore, MDM2 inhibitors typically decrease Snail protein levels and suppress migration, invasion, and metastasis, even in a p53-independent manner [42,43,44,45].

Thus, inhibiting MDM2 seems to be an attractive strategy for suppressing EMT, regardless of p53 status (wild-type, null, or mutant). Consistent with this, our current research demonstrated that MDM2 knockdown significantly down-regulates Snail protein levels in A549 cells. Furthermore, *Z. nitidum* extract significantly reduced Snail protein levels.

Based on the spectroscopic data of the ethanol extract of *Z. nitidum*, we identified several benzophenanthridine alkaloids, including 8-acetonyldihydrochelerythrine, nitidine, terihanine, and sanguinarine. These alkaloids have previously been isolated from this species [53,66,67].

*Z. nitidum*-mediated stabilization of both p53 and MDM2 is consistent with our docking analysis data. 8-Acetonyldihydrochelerythrine, nitidine, terihanine, and sanguinarine shared several key interacting residues with the reference inhibitor, Nutlin-3a. These residues include Leu54, Ile61, Val93, Ile99, and His96. They are located within the p53-binding cleft of MDM2. Notably, sanguinarine formed π–cation and π–alkyl interactions similar to those of nutlin-3a. Compounds 8-acetonyldihydrochelerythrine and nitidine, on the other hand, established additional hydrogen bonds with His96, enhancing their stability within the pocket. The overlap in binding residues and interaction types suggests that these benzophenanthridine alkaloids may inhibit MDM2 by occupying the same hydrophobic groove as nutlin-3a. This could potentially block p53 degradation and contribute to p53 stabilization in NSCLC. 

Sanguinarine exhibits a wide range of biological activities, primarily due to its potent anticancer properties, which include inhibiting proliferation and inducing apoptosis and autophagy. It exhibits remarkably low IC_50_ values (in the range of several μM) and demonstrates strong efficacy against multiple types of malignancies by targeting the JAK/STAT, MAPK, and survival signaling pathways, as well as Ca^2+^-activated Cl^−^ channels (TMEM16A) [68,69]. Although it displayed the best cytotoxicity against NSCLC cell lines in our studies and can be considered to have high anticancer potential, we did not focus on this compound in the present research because it neither affects the MDM2–p53 axis nor impacts the energy metabolism of our NSCLC cell models.

The biological properties of 8-acetonyldihydrochelerythrine are largely unexplored. However, it has demonstrated notable antiproliferative activity against human breast cancer cells (HCC 1395, IC_50_ = 9.99 μg/mL), with a favorable selectivity index (SI = 4.79) [70]. This is the first report of its anticancer potential, establishing it as a promising lead alkaloid.

Unlike other benzophenanthridine alkaloids, terihanine has rarely been reported in the literature, and its pharmacological profile remains largely unexplored. To date, no studies have described its anticancer or cytotoxic properties. Thus, the present work may be one of the first attempts to investigate this compound’s biological potential. Although terihanine did not exhibit proper cytotoxicity, it significantly stabilized MDM2 and p53, similar to nutlin-3a, a well-known MDM2 inhibitor. This suggests that terihanine could be considered a lead compound for the pharmacological inhibition of the MDM2-p53 axis. Notably, it also strongly stabilized MDM2 in p53-null H1299 cells. It is possible that terihanine inhibits the ubiquitin ligase activity of MDM2, leading to its accumulation, as is the case with MEL23 and MEL24 [71]. Furthermore, it significantly decreases Snail protein levels in both p53-positive and p53-negative NSCLC cells.

Our phytochemical and mechanistic investigations revealed that nitidine, a benzophenanthridine alkaloid, was the most superior constituent. It induced p53 stabilization and MDM2 downregulation, which led to the inhibition of proliferation, migration, and invasion, as well as the dramatic suppression of energy metabolism, in both p53+ and p53-null NSCLC cells. Unlike terihanine, benchmark MDM2 inhibitor, nutlin-3a, and most other MDM2 inhibitors, which induce MDM2 stabilization in addition to p53, nitidine induces a strong decrease in MDM2 protein levels despite the 10-fold increase in MDM2 mRNA levels induced by p53 as its transcriptional target. Presumably, nitidine induces MDM2 autoubiquitination, as with SP141, the only MDM2-targeting molecule known thus far, which decreases MDM2 protein levels independently of p53 [22]. Using the CHX pulse assay (Figure 9), we found that nitidine promoted MDM2 degradation. Addition of MG132, a proteasome inhibitor, reversed nitidine’s negative effect on the MDM2 protein level (Figure 10). Similarly to SP141, we observed nitidine-mediated dramatic suppression of the MDM2 protein level in both p53-positive and p53-negative cells, which is valuable for potential therapeutic applications. Although nitidine has a similar impact on both p53+ and p53-null NSCLC cell lines, it is more efficient in A549 cells that express wild-type p53.

Thus, although molecular docking predicted similar MDM2 binding affinities for the tested compounds, we observed no strong correlation with their cellular effects. This discrepancy likely reflects distinct mechanisms involved in the modulation of MDM2 functions (e.g., disruption of the MDM2-p53 interaction, inhibition of MDM2 ubiquitin ligase activity, or MDM2 destabilization), as well as compound-specific differences in permeability, off-target effects, and metabolic stability. Sanguinarine, despite its good docking score, may fail to phenocopy MDM2 inhibition due to its well-documented pleiotropic effects on multiple cellular processes. Another contributing factor to this may be the incomplete understanding of which docking parameters most accurately predict binding affinity to MDM2. Thus, the lack of orthogonal validation (e.g., SPR, ITC, microscale thermophoresis, or competitive binding against Nutlin-3a) is a limitation of our current study. Consequently, while docking alone suggests a potential interaction with MDM2 that warrants further investigation, the observed effects on various molecular processes (DNA damage, energy metabolism, signaling pathways, oncogene expression, etc.) may either be MDM2-dependent or -independent.

It is important to note that, while nutlins proved the concept of pharmacologically targeting the MDM2–p53 interaction, they were not clinically viable for a variety of reasons. These molecules are designed to affect only malignancies with wild-type p53 (p53WT) [27]. This excludes over 50% of cancer patients whose tumors carry p53 mutations. Furthermore, nutlin application selects for multidrug-resistant cancer cells harboring TP53 mutations [72]. Additionally, by blocking the p53–MDM2 interaction, these inhibitors disrupt the negative feedback loop in which p53 induces MDM2 expression. This can lead to an increase in MDM2 protein levels, which may counteract the drug’s effect or promote other p53-independent oncogenic MDM2 functions [27].

In contrast, MDM2 degradation inducers such as SP141 and PROTACs are generally more effective and target malignancies regardless of their p53 status [27]. This approach seems more promising than developing inhibitors of MDM2–p53 interactions. This adds to the case for nitidine as a compound that dramatically decreases MDM2 protein levels.

Consistent with the aforementioned impact of MDM2 on Snail and EMT, we observed a nitidine-dependent decrease in Snail and β-catenin protein levels, as well as dramatic suppression of migratory and invasive capabilities.

It is noteworthy that although the phenotypic effects of *Z. nitidum* extract and nitidine are similar (strong suppression of p53+ and p53- NSCLC cell lines), their molecular effects differ. While *Z. nitidum* extract led to MDM2 accumulation, nitidine dramatically decreased MDM2 protein levels and suppressed both respiration and glycolysis. These features are highly desirable for potential therapeutic applications. In other words, the effects of nitidine are superior to the combined effects of the different compounds present in *Z. nitidum* extract.

In general, nitidine exhibits significant anticancer properties across various malignancies by inhibiting crucial cellular pathways [73]. In breast cancer, nitidine suppresses metastasis by inhibiting matrix metalloproteinases (MMPs) and the platelet-derived growth factor (PDGF) pathway [74]. It also reverses epithelial–mesenchymal transition by blocking Hedgehog signaling [75]. In ovarian cancer, nitidine induces apoptosis through the Akt and Fas pathways, as well as inhibits proliferation by downregulating Skp2 [76]. Nitidine suppresses EMT and stem cell-like properties in glioblastoma by inhibiting JAK2/STAT3 signaling [77]. In lung and gastric cancers, nitidine exerts its effects by downregulating NEDD4, activating the Hippo pathway, and inhibiting the STAT3/miR-17-5p axis [78].

Figure 7 and data from the literature indicate that nitidine triggers a DNA damage response in both p53-dependent and -independent manners. In cervical cancer, it induces DNA damage signaling (γH2AX) and Chk2 activation, which elicits p53/Bim-dependent apoptosis [38]. In hepatocellular carcinoma, nitidine induces G2/M cell cycle arrest by upregulating the p53/14-3-3 sigma/CDK1 axis [79]. Recently, Voukeng et al. demonstrated that nitidine exerts its anticancer activity by intercalating DNA, inhibiting topoisomerases I and II, and triggering DNA damage. This subsequently disrupts nucleolar integrity and represses ribosome biogenesis by destabilizing RNA polymerase I recruitment [49]. Therefore, we propose that both MDM2 destabilization and DNA damage may exert a cumulative effect on p53 stabilization.

Taken together, our results combined with the literature data suggest that nitidine chloride has multiple anticancer properties. It induces p53 stabilization through a DNA damage-dependent mechanism and promotes the inhibition of MDM2 ubiquitin ligase. Furthermore, its negative impacts on EMT factors, bioenergetics, migration and invasion capabilities, and stem cell-like properties add to its potential therapeutic value.

## 4. Materials and Methods

### 4.1. Plant Materials

A total of 31 plant samples were collected at the end of May 2024 in Suoi Hai commune, Hanoi, Vietnam. All voucher specimens were authenticated and subsequently deposited at the Center for Natural Products Development and Technology—Equipments, Institute of Chemistry, Vietnam Academy of Science and Technology (VAST), Vietnam. The voucher specimen C-848 (*Zanthoxylum nitidum* (Roxb.) DC.) has been deposited in the Herbarium of the Institute of Chemistry, VAST.

### 4.2. Cell Lines and Reagents

The NSCLC cell lines used in this study (A549 and H1299) were purchased from ATCC (Manassas, VA, USA). The cells were cultured in RPMI culture medium containing 10% fetal bovine serum, 0.05 mg/mL gentamycin, and 2 mM L-glutamine at 37 °C in a 5% CO_2_ atmosphere.

H1299 cells with TetOn-induced expression of exogenous p53 were a kind gift of Dr. Patricia Muller [80] and A549 cells with MDM2 knockdown were described in [47] and [23], respectively. Tetracycline hydrochloride was purchased from Gibco (A39246). Nutlin-3a (SML0580) was purchased from Sigma (Burlington, MA, USA).

### 4.3. Apparatus

^1^H-NMR (600 MHz) and ^13^C-NMR (150 MHz) spectra were recorded on a Bruker Advance 600 MHz spectrometer. The HR-ESI-MS were obtained from a Varian FT-MS spectrometer and MicroQ-TOF III (Bruker Daltonics, Bremen, Germany). Column chromatography was performed on silica gel (Si 60 F254, 230–400 mesh, Merck) and HP-20 Diaion (Mitshubishi Chemical, Tokyo, Japan). All solvents were redistilled prior to use. Precoated TLC plates (Si 60 F 254) were used for analytical purposes. Compounds were visualized under UV radiation (254, 365 nm) and by spraying plates with 10% H_2_SO_4_, followed by heating with a heat gun.

### 4.4. Extraction and Isolation

For extraction, the air-dried and powdered plant materials were processed following the same general procedure. Specifically, the air-dried, powdered stems and twigs of *Z. nitidum* (2.5 kg) were extracted four times with 80% ethanol (4 × 5 L) at room temperature. The combined extracts were evaporated under reduced pressure to afford 132.5 g of a thick ethanolic extract. All other plant samples were treated using the same extraction procedure.

The ethanol extract was divided into two portions. The first portion was subjected to liquid–liquid partition with dichloromethane, yielding the dichloromethane fraction (ZD, 28.3 g) and the remaining aqueous layer (ZW). The second portion was acidified with 5% HCl to pH 1–2, filtered to remove insoluble residues, and the resulting solution was extracted with dichloromethane to obtain the Dichlo fraction (Z2D, 14.2 g). The aqueous layer was then neutralized with 5% NaOH to pH 9–10 and further partitioned with ethyl acetate to afford the EtOAc fraction (Z2E, 28.7 g). The final aqueous residue after extraction was designated as Z2W (7.8 g).

Fraction ZD (28.3 g) was subjected to column chromatography and eluted with a gradient solvent system of hexane–acetone (12:1 → 1:1, *v*/*v*), followed by acetone and methanol washing to obtained fourteen subfractions, ZDA–ZDP. Subfraction ZDB (5.8 g) was further subjected to column chromatography and eluted with a gradient of hexane–dichloromethane (12:1 → 1:1, *v*/*v*), followed by dichloromethane–methanol (5:1 → 1:1, *v*/*v*), and finally washed with methanol. Sixteen subfractions were obtained, ZDB1-ZDB16. Subfraction ZDB9 (400 mg) was subjected to column chromatography and eluted with a gradient of dichloromethane–acetone (8:1 → 1:1, *v*/*v*), affording 14 subfractions, ZDB9A-ZDB9O. Subfraction ZDB9K was further purified on a Mini-C column using hexane–acetone (8:1, *v*/*v*) as the eluent to yield compound **1** (20 mg).

A portion of fraction Z2E (11.88 g) was subjected to column chromatography and eluted with a gradient of dichloromethane–methanol (8:1 → 1:1, *v*/*v*), followed by methanol washing, to afford 13 subfractions, Z2E1-Z2E13. Subfractions Z2E5 (428 mg) and Z2E6 (1.51 g) were combined and subjected to column chromatography using dichloromethane–methanol (5:1 → 1:1, *v*/*v*), followed by methanol washing, affording 12 subfractions. Subfraction Z2E5B (645 mg) was then purified by column chromatography with dichloromethane–methanol (20:1 → 1:2, *v*/*v*) and methanol washing, yielded compound **2** (21 mg). Subfraction Z2E10 (421 mg) was further chromatographed using dichloromethane–methanol (20:1 → 1:2, *v*/*v*) and finally washed with methanol, yielding 10 subfractions. Subfraction 6 was washed with methanol to obtained compound **6** (5.1 mg). 

Fraction Z2W (7.8 g) was subjected to column chromatography and eluted with a gradient of dichloromethane–methanol (15:1 → 1:1, *v*/*v*), followed by methanol washing, to afford 14 subfractions. Subfractions Z2W7 (476 mg) and Z2W9 (532 mg) were combined and further chromatographed using dichloromethane–methanol–ammonia (10:1:0.1 → 2:1:0.1, *v*/*v*) as the eluent, followed by methanol washing, yielding compound **3** (4.9 mg) and compound **4** (20 mg).

Fraction ZDD (4.8 g) was subjected to column chromatography and eluted with a gradient of hexane–ethyl acetate (5:1 → 1:1, *v*/*v*), followed by ethyl acetate–methanol (5:1 → 1:1, *v*/*v*), and finally washed with methanol, affording nine subfractions ZDD1-ZDD9. Subfractions ZDD2 and ZDD3 were combined (1.23 g) and further chromatographed using a solvent system of hexane–ethyl acetate (10:1 → 1:5, *v*/*v*), followed by ethyl acetate–acetone (5:1 → 2:1, *v*/*v*), and washed with methanol to yield seven subfractions, ZDD2A-ZDD2G. Subfraction ZDD2D (100 mg) was further purified on a mini column using hexane–ethyl acetate (10:1, *v*/*v*) as a single solvent system, affording compound **5** (103.5 mg).

### 4.5. MTT Assay

For the MTT assay, 10,000 cells were seeded in each well of a 96-well plate overnight. Ten replicate wells were prepared for each experimental condition. After incubating for 24 h, the cells were treated with *Z. nitidum* extract and nitidine at the indicated concentrations for 48 h. Cells treated with EtOH or DMSO were used as a control. Then, 10 μL of Thiazolyl Blue solution (5 mg/mL, Paneko, Russia) was added to each well. The plates were then incubated for 3.5 h at 37 °C in a CO_2_ incubator. Then, the medium containing thiazolyl blue was aspirated and the resulting formazan crystals were solubilized by adding 150 μL of isopropyl alcohol containing 40 mM HCl and 0.1% NP-40. Absorbance was measured at 570 nm with 630 nm as the reference wavelength using a Bio-Rad iMark microplate reader (Bio-Rad, Hercules, CA, USA). Data are presented as mean ± SD.

### 4.6. Real-Time PCR

Total RNA was isolated from cells using a TRIzol-based reagent according to the manufacturer’s protocol. cDNA synthesis was performed using 3 μg of total RNA, oligo d(T) primers, and a RevertAid First-Strand cDNA Synthesis Kit. Real-time PCR amplification was carried out in triplicate on a CFX1000 thermal cycler (Bio-Rad, Hercules, CA, USA) using a SYBR Green Master Mix (Evrogen, Moscow, Russia). Data analysis was conducted using the CFX Manager software, with β-actin serving as the internal reference gene. Relative gene expression levels were determined using the 2^−ΔΔCt^ method. Primer sequences are provided in Table S2.

### 4.7. Western Blot

Cell lysates were prepared using RIPA buffer (150 mM NaCl, 50 mM Tris-HCl [pH 7.5], 0.3% Triton X-100, and 1 mM PMSF, supplemented with a protease inhibitor cocktail) and then sonicated. Protein concentrations were determined using a BCA assay kit (Thermo Scientific, Waltham, MA, USA), and the samples were normalized with Laemmli buffer. An equal amount of protein (30 μg) was separated by 13% SDS-PAGE in Tris-glycine running buffer and transferred to a PVDF membrane (Bio-Rad, Hercules, CA, USA). The membranes were blocked with 5% nonfat milk in PBST and subsequently incubated overnight at 4 °C with the following primary antibodies: SNAI1 (K002369P, Solarbio, Beijing, China); β-catenin (sc-7963, Santa Cruz Biotechnology, Dallas, TX, USA); P53 (OP43-100UG, EMD Millipore Corporation, Billerica, MA, USA); MDM2 (OP115-100UG, EMD Millipore Corporation, Billerica, MA, USA); GAPDH (GB11002-100, Servicebio, Wuhan, China); and β-actin (#8457, Cell Signaling Technology, Danvers, MA, USA). After washing with PBST, the membranes were incubated with HRP-conjugated anti-mouse or anti-rabbit secondary antibodies (1:10,000; Sigma-Aldrich, St. Louis, MI, USA). Protein bands were visualized using an ECL detection system (Thermo Fisher Scientific, Waltham, MA, USA) and captured using a ChemiDoc™ Touch Imaging System (Bio-Rad, Hercules, CA, USA). The ImageJ software (version 1.51) was used to analyze the data, and protein expression levels were normalized to β-actin and GAPDH. Data are presented as the mean ± SEM from three independent biological experiments.

### 4.8. Cycloheximide Pulse-Chase Assay

To assess the degradation rate of MDM2 and p53 proteins, cells were treated with nitidine (25 μM) for 24 h, followed by the addition of cycloheximide (CHX, 100 μg/mL) to block new protein synthesis. Cells were harvested at the indicated time points (0, 1, 1.5, and 2 h) and lysed for protein quantification and Western blot analysis.

### 4.9. MG132 Protein Stability Chase Assay

To determine whether nitidine induces MDM2 degradation by the ubiquitin-proteasome pathway, cells were treated with 25 μM nitidine for 16 h followed by the addition of the proteasome inhibitor MG132 (10 μM) for 8 h. Then, cells were harvested and lysed for protein quantification and Western blot analysis. Stabilization of the target protein upon MG132 co-treatment, compared to nitidine alone, confirms its degradation via the ubiquitin–proteasome system.

### 4.10. Cell Cycle Analysis

Twenty-four hours after seeding, the cells were exposed to *Z. nitidum* at concentrations of 0, 25, 50, or 75 μg/mL for 24 h in triplicate. After treatment, the cells were collected, washed with PBS, and fixed in 70% ethanol at −20 °C for one hour. The DNA content was evaluated by staining the cells with 50 μg/mL propidium iodide (PI; Abcam, Waltham, MA, USA) and 1 μg/mL RNase A (Thermo Scientific, Waltham, MA, USA) for thirty minutes. Flow cytometric analysis was performed using a CytoFLEX (Beckman Coulter, Carlsbad, CA, USA), and the data were analyzed using the CyteExpert software (Beckman Coulter, Carlsbad, CA, USA). A Student’s *t*-test was used to determine statistically significant pairwise differences. Data are presented as mean ± SEM.

### 4.11. Apoptosis and Total Cell Death

To assess the impact of *Z. nitidium* on cell viability and apoptosis, cells were incubated with Z. nitidium (0, 25, 50, or 75 μg/mL) for 24 h, followed by staining with Annexin V-FITC/7-AAD (Thermo Fisher Scientific, Waltham, MA, USA) according to the manufacturer’s instructions. Flow cytometric analysis was performed using a CytoFLEX instrument (Beckman Coulter, Carlsbad, CA, USA), with at least 5000 events acquired per sample in the appropriate fluorescence channels. Three independent experiments were conducted, and median fluorescence values were used for quantification. A Student’s *t*-test was used to determine statistically significant pairwise differences. Data are presented as mean ± SEM from three independent experiments.

### 4.12. Reactive Oxygen Species (ROS) Measurement

Intracellular ROS levels were assessed using the cell-permeable fluorogenic probe DCFDA (2′,7′-dichlorofluorescein diacetate). After 24 h treatment with nitidine, cells were harvested, washed once with PBS, and incubated with 10 μM DCFDA in serum-free medium for 30 min at 37 °C in the dark. Cells were then washed twice with PBS, resuspended in PBS, and immediately analyzed on a CytoFLEX flow cytometer (Beckman Coulter). For each sample, at least 10,000 events were acquired. The median fluorescence intensity (MFI) was recorded using the Cytexpert software (Beckman Coulter). Data are presented as fold change relative to the control (DMSO-treated cells). Statistical significance was determined using Student’s *t*-test.

### 4.13. Autophagy Assay

Autophagy was evaluated using an Autophagy Assay Kit (Fluorescent) (ab139484, Abcam) according to the manufacturer’s instructions. This kit employs a fluorescent dye that selectively labels autophagic vacuoles. Following 24h treatment with nitidine, cells were harvested, washed with PBS, and stained with the autophagy detection reagent for 30 min at 37 °C in the dark. Cells were then washed and resuspended in assay buffer. Fluorescence was measured using a CytoFLEX flow cytometer (Beckman Coulter), and at least 10,000 events were recorded per sample. The median fluorescence intensity (MFI) was quantified using the Cytexpert software. The level of autophagy is expressed as fold change over the control (DMSO-treated cells). All experiments were performed in triplicate, and statistical analysis was carried out using Student’s *t*-test.

### 4.14. Immunofluorescence

Cells were seeded on coverslips in 24-well plates and allowed to attach overnight. The next day, cells were treated with 40 μM nitidine or 40 μM etoposide (positive control) for 4 h. DMSO-treated cells served as a negative control. After treatment, cells were washed twice with PBS, fixed with 4% paraformaldehyde for 15 min at room temperature, and permeabilized with 0.5% Triton X-100 in PBS for 10 min. Non-specific binding was blocked with 5% bovine serum albumin (BSA) in PBS for 1 h. Subsequently, cells were incubated overnight at 4 °C with a primary antibody against γH2AX (dilution 1:500, e.g., Millipore, #05-636). After three washes with PBS, cells were incubated with an Alexa Fluor-conjugated secondary antibody (1:1000, e.g., anti-rabbit Alexa Fluor 564) for 1 h at room temperature in the dark. Nuclei were stained with DAPI (1 μg/mL) for 5 min. Coverslips were mounted onto glass slides using an anti-fade mounting medium. Images were acquired using a confocal microscope (Olympus FV3000) with a 40× oil immersion objective. The intensity of pan-nuclear γH2AX staining was quantified using the ImageJ software (NIH, Bethesda, MD, USA) from at least three independent fields per condition. Data are presented as mean ± SEM, and statistical significance was determined using Student’s *t*-test.

### 4.15. SeaHorse Energy Profiling

We used the Seahorse XFe24 Analyzer (Agilent, Santa Clara, CA, USA) to accurately quantify glycolysis and respiration. Cells were seeded at a density of 30,000 cells per well in XFe24 cell culture microplates, with five replicates. The next day, the cells were treated with the following test substances at their final concentrations: *Z. nitidum* ethanol extract (25–75 μg/mL), 8-acetonyldihydrochelerythrine (8-AC) (100 μM), nitidine (40 μM), terihanine (100 μM), and sanguinarine (1.7 μM), for 24 h. Ethanol or DMSO was used as the control. On the day of analysis, we used the Seahorse XF Real-Time ATP Rate Assay Kit (Agilent, Santa Clara, CA, USA) according to the manufacturer’s protocol. The final concentrations of the oligomycin and rote-none/antimycin A mix were 3 µM each. Glycolytic and mitochondrial ATP production rates were quantified using the Seahorse XF Real-Time ATP Rate Assay Report Generator. A Mann–Whitney U-test was applied to check for statistically significant differences.

### 4.16. Migration and Invasion Assays

For the Transwell migration and invasion assays, 500,000 cells were seeded in a serum-free medium in the upper chamber of an 8 μm porous insert. A 20% serum solution was placed in the lower chamber. Nitidine (40 μM) or DMSO (control) was added immediately. For the invasion assay, the membrane was coated with Matrigel prior to cell seeding. The cells were allowed to migrate through the pores into the lower chamber for 24 h. Non-migrated cells were then removed from the top. The migrated cells on the underside of the membrane were fixed and stained with Crystal Violet in 4% PFA for 15 min. After washing, the cells were counted under a microscope for quantification. Three biological replicates were used. In a parallel experiment, trypan blue staining was used to assess viability. Finally, the number of migrated/invaded cells was normalized to the number of viable cells. A Student’s *t*-test was used to determine statistically significant pairwise differences. Asterisks represent significant differences (* *p* ≤ 0.05; ** *p* ≤ 0.01).

For the wound-healing assay, the monolayer cells were scratched with a 200 μL pipette tip. Then, the cells were washed with PBS and the media were changed to remove the detached cells. Three biological replicates were used. After photographing the cells, nitidine (40 μM) or DMSO (control) was added for 24 h. Finally, the cells were photographed again, and the rate of wound closure was calculated. In parallel experiment, trypan blue staining was used for the assessment of viability. Finally, the rate of wound closure was normalized to the number of viable cells. A Student’s *t*-test was used to determine statistically significant pairwise differences. Asterisks represent significant differences (* *p* ≤ 0.05; ** *p* ≤ 0.01).

### 4.17. Molecular Docking

The benzophenanthridine alkaloids isolated from *Z. nitidum* were subjected to molecular docking to investigate their potential inhibitory activity against the ubiquitin ligase function of the MDM2 protein using AutoDock Vina version 1.2.7 [81,82]. The crystal structure of MDM2 was retrieved from the RCSB Protein Data Bank, selecting the form co-crystallized with a ligand in the p53 binding site to accurately define the active pocket (PDB ID: 4HG7) [83]. The protein was prepared by removing water molecules and co-crystallized ligands, adding hydrogens, assigning Kollman charges, and saving in PDBQT format using the MGLTools software. The benzophenanthridine alkaloids were drawn in the ChemDraw software, followed by geometric optimization using the MMFF94 force field and conversion to PDBQT format in the OpenBabel software. The search space was defined around the binding site of the natural ligand or the p53 interaction region, with a grid box size of approximately 24 × 24 × 24 Å and the center coordinates determined based on the co-crystallized ligand. Docking simulations were performed using the default parameters of the AutoDock Vina program, with the exhaustiveness value set to 400, the number of generated conformations (num_modes) set to 20, and the energy difference between the best poses (energy_range) set to 3 kcal/mol. The docking calculations were executed on a multi-core CPU to accelerate processing. After docking, the conformations with the lowest binding free energies (kcal/mol) were selected and converted to PDB format for visualization in the PyMOL software. The interactions between the ligands and MDM2 were further analyzed using the Discovery Studio Visualizer software to identify hydrogen bonds, π–π stacking, and hydrophobic interactions with key amino acid residues in the binding pocket.

### 4.18. Co-Immunoprecipitation

A549 cells expressing HA-tagged MDM2 (described in [23]) were treated with 40 μM nitidine, 10 μM nutlin-3a, or DMSO (control) for 16 h, followed by co-treatment with MG132 (10 μM) for an additional 6 h. Cells were lysed, and 500 μg of protein lysate was incubated overnight at 4 °C with either anti-HA antibody (10 μg/mL) or, as a negative control, normal rabbit IgG (10 μg/mL) at the same concentration. The next day, Protein G-Sepharose beads were added to each sample and incubated for 4 h at 4 °C. The immunoprecipitated complexes were washed three times, eluted by boiling in Laemmli buffer, and analyzed by Western blot. Quantification was performed using the ImageJ software, and the MDM2-p53 interaction level was normalized to the control (DMSO-treated cells).

### 4.19. Statistical Analysis

We employed Student’s *t*-test to identify pairwise statistically significant differences in experiments with *n* = 3. The Mann–Whitney U-test was utilized for the SeaHorse energy profiling and MTT assays with sample sizes n = 5 and n = 6, respectively. The analysis was conducted using GraphPad Prism version 8. A statistical difference was established at *p* < 0.05. * *p* < 0.05; ** *p* < 0.01; *** *p* < 0.001; nonsignificant, ns.

## 5. Conclusions

Our findings identify *Z. nitidum* and nitidine as promising sources for developing novel MDM2-targeting therapeutics against NSCLC irrespective of the p53 status.

## Figures and Tables

**Table 1:** Binding affinities and interaction profiles of studied benzophenanthridine alkaloids from *Z. nitidum* with the MDM2 protein compared to the reference compound Nutlin-3a.

Compound	Binding Affinity (kcal/mol)	Amino Acid Residues	Interaction Type
8-acetonyldihydrochelerythrine (**1**)	−8.304	His96	Hydrogen bond
		Leu54	π-alkyl
		Gly58	π-σ
Nitidine (**2**)	−8.106	Ile61, Leu54, Val93	π-alkyl
		His96	Hydrogen bond
Terihanine (**3**)	−8.491	Leu94	Amide-π stacked and π-alkyl
		His96	π-σ
Sanguinarine (**4**)	−9.187	Ile61, Val93, Ile99	π-alkyl
		Leu54	π-σ
		His96	π-cation
Reference compound Nutlin-3a	−7.786	Val93, Leu54, His96, Tyr100, Phe91, Ile99, Ile61, Leu57	Alkyl and π-alkyl
		His96	π-π stacked
		Val93	π-σ

**Table 2:** Cytotoxic and cytostatic activity of compounds isolated from *Zanthoxylum nitidum* in NSCLC cell lines.

No	Compound	IC_50_ (µM)		
		A549	H460	HI299
**1**	8-acetonyldihydrochelerythrine	115 ± 13	105 ± 16	>200
**2**	Nitidine	18 ± 1.5	20 ± 2.2	25 ± 3.1
**3**	Terihanine	82 ± 7.5	160 ± 12	>200
**4**	Sanguinarine	1.5 ± 0.2	1.6 ± 0.1	2.1 ± 0.2
**5**	Sesamin	125 ± 9	>200	>200

## Data Availability

Data is contained within the article and the Supplementary Material.

## Supplementary Materials

The following supporting information can be downloaded at: https://www.mdpi.com/article/10.3390/ph19060814/s1. Table S1. The results of screening of 33 Vietnamese ethnomedicinal plant extracts for their anticancer properties and selectivity in NSCLC and non-cancerous cell lines. MTT data. Table S2. List of primers used for real-time PCR. Figure S1. The impact of MDM2 knockdown on the protein levels of p53 and Snail in A549 cell line. The diagrams show the results of quantification performed using the Image J software. Student’s *t*-test was used to determine pairwise statistically significant differences. Asterisks represent a significant difference in protein expression (*** *p* ≤ 0.001). Figure S2. 1H-NMR spectrum of 8-acetonyldihydrochelerythrine (1). Figure S3. 13C-NMR spectrum of 8-acetonyldihydrochelerythrine (1). Figure S4. DEPT spectrum of 8-acetonyldihydrochelerythrine (1). Figure S5. HR-ESI-MS spectrum of 8-acetonyldihydrochelerythrine (1). Figure S6. 1H-NMR spectrum of nitidine (2). Figure S7. 13C-NMR spectrum of nitidine (2). Figure S8. HR-ESI-MS spectrum of nitidine (2). Figure S9. 1H-NMR spectrum of terihanine (3). Figure S10. HR-ESI-MS spectrum of terihanine (3). Figure S11. 13C-NMR spectrum of terihanine (3). Figure S12. 1H-NMR spectrum of sanguinarine (4). Figure S13. 13C-NMR spectrum of sanguinarine (4). Figure S14. DEPT spectrum of sanguinarine (4). Figure S15. HR-ESI-MS spectrum of sanguinarine (4). Figure S16. 1H-NMR spectrum of sesamin (5). Figure S17. HR-ESI-MS spectrum of sesamin (5). Figure S18. 1H-NMR spectrum of myo-inositol (6). Figure S19. HR-ESI-MS spectrum of myo-inositol (6). Figure S20. Nitidine interferes with MDM2-p53 binding. Western-blot demonstrates the results of co-immunoprecipitation of HA-tagged MDM2 with endogenous p53 in DMSO- (control) and nitidine-treated A549 cells. Nutlin-3a was used as a positive control. Nit—nitidine, Nut—nutlin-3a, IgG—control antibody. The diagrams show the results of quantification performed using the ImageJ software. Student’s *t*-test was used to determine pairwise statistically significant differences. Asterisks represent a significant difference in protein expression (* *p* ≤ 0.05; ** *p* ≤ 0.01). Figure S21. Nitidine affects the protein levels of p53, MDM2, Snail, and β-catenin in H1975 cell line bearing mutant p53. Western-blot. The diagrams show the results of quantification performed using the Image J software. Student’s *t*-test was used to determine pairwise statistically significant differences. Asterisks represent a significant difference in protein expression (* *p* ≤ 0.05; ** *p* ≤ 0.01). Figure S22. The impact of nitidine treatment (40 μM, 24 h) on wound closure in A549 and H1299 cell lines. Wound-healing assay. In parallel experiment, trypan blue staining was used for the assessment of viability. Finally, the rate of wound closure was normalized to the number of viable cells. A Student’s *t*-test was used to determine statistically significant pairwise differences. Asterisks represent significant differences (* *p* ≤ 0.05; ** *p* ≤ 0.01).

## Abbreviations

NSCLC	Non-small cell lung cancer
EMT	Epithelial–mesenchymal transition
NC	Nitidine
TC	Terihanine
SC	Sanguinarine
8AC	8-acetonyldihydrochelerythrine
ROS	Reactive Oxygen Species
CMX	Cyclocheximide
