# Supplementary material for: Discovery of Benzophenanthridine Alkaloids from Zanthoxylum nitidum That Target the MDM2–p53 Axis in NSCLC
Source: Pharmaceuticals (Basel). 2026 May 22;19(6):814. doi: 10.3390/ph19060814 (PMC13304904; doi:10.3390/ph19060814)
Supplement: Supplementary file 1 [file pharmaceuticals-19-00814-s001.zip › tables.pdf]

Table S1. The results of screening of 33 Vietnamese enthnomedicinal plant extracts for their anticancer properties and selectivity in NSCLC and non-cancerous cell lines. MTT data.

No.	Internal number	Species*	Vietnamese local name	Plant material and extract	Collected location	IC 50 values (μg/mL)				
						NSCLC cell lines			Non-cancerous cell lines	
						A549	H460	H1299	DF2	FRSN
1	VN-82	*Crotalaria* sp.	Mất ngủ	Leaf and branch Ethanol 80% extract	Hanoi	> 300	> 300	> 300	n.d.	n.d.
2	VN-83	*Hovenia dulcis* Thunb.	Khúng khéng	Ethanol extract 70%	Cao Bang Province	> 300	> 300	> 300	n.d.	n.d.
3	VN-84	*Hovenia dulcis* Thunb.	Khúng khéng	Fruit Water 100% fraction DIAION	Cao Bang Province	> 300	> 300	> 300	n.d.	n.d.
4	VN-91	*Strobilanthes* sp.	Chàm tía	Leaf and stem EtOAc fraction	Cao Bang Province	> 300	> 300	> 300	n.d.	n.d.
6	VN-32	*Morinda longissima* Y.Z.Ruan	Nhó đông	Root EtOH 80% extract	Son La Province	> 300	> 300	> 300	n.d.	n.d.
5	VN-33	*Morinda longissima* Y.Z.Ruan	Nhó đông	Root Water extract	Son La Province	> 200	> 200	> 200	n.d.	n.d.
6	VN-34	*Glochidion eriocarpum* Champ. ex Benth.	Lồ lào nhông	Leaves and branches EtOH 80% extract	Suoi Hai, Hanoi	> 300	> 300	> 300	n.d.	n.d.
8	VN-35	Unidentified	Rào kía	Stems EtOH 80% extract	Suoi Hai, Hanoi	> 200	> 200	> 200	n.d.	n.d.
7	VN-36	*Ventilago leiocarpa* Benth.	Tầm khá	Stems EtOH 80% extract	Suoi Hai, Hanoi	> 300	> 300	> 300	n.d.	n.d.
8	VN-37	*Derris marginata* (Roxb.) Benth.	Sâm Nam	Stems EtOH 80% extract	Suoi Hai, Hanoi	> 200	> 200	> 200	n.d.	n.d.

9	VN-38	*Atalantia guillauminii* Swingle	Quýt rừng	Leaves and branches EtOH 80% extract	Suoi Hai, Hanoi	> 200	> 200	> 200	n.d.	n.d.
10	VN-39	*Lasia spinosa* (L.) Thwaites	Hậu gài khím	Rhizomers EtOH 80% extract	Suoi Hai, Hanoi	> 200	> 200	> 200	n.d.	n.d.
11	VN-40	*Antidesma tonkinense* Gagnep.	Lồ lào duôi	Stems EtOH 80% extract	Suoi Hai, Hanoi	> 200	> 200	> 200	n.d.	n.d.
12	VN-41	*Sarcandra glabra* (Thunb.) Nakai	Sói rừng	Leaves and branches EtOH 80% extract	Suoi Hai, Hanoi	124 ± 10	180 ± 16	122 ± 12	>300	>300
13	VN-42	*Antidesma bunius* (L.) Spreng.	Lồ lào búng	Stems EtOH 80% extract	Suoi Hai, Hanoi	> 200	> 200	> 200	n.d.	n.d.
14	VN-43	*Pottsia laxifora* Kuntze	Dây cao su	Stems EtOH 80% extract	Suoi Hai, Hanoi	157 ± 14	149 ± 12	180 ± 17	>300	>300
15	VN-44	*Fissistigma chloroneurum* (Hand.-Mazz.) Chun	Cù Biệt	Stems EtOH 80% extract	Suoi Hai, Hanoi	> 200	> 200	> 200	n.d.	n.d.
16	VN-45	*Homonoia riparia* Lour.	Xùi liền	Leaves and branches EtOH 80% extract	Suoi Hai, Hanoi	> 200	> 200	> 200	n.d.	n.d.
17	VN-46	Unidentified	Năm lá	Stems EtOH 80% extract	Suoi Hai, Hanoi	> 200	> 200	> 200	n.d.	n.d.
18	VN-47	Unidentified	Cây đùi	Stems EtOH 80% extract	Suoi Hai, Hanoi	> 200	> 200	> 200	n.d.	n.d.
19	VN-48	Unidentified	Xà tồng	Stems EtOH 80% extract	Suoi Hai, Hanoi	> 200	> 200	> 200	n.d.	n.d.
20	VN-49	Unidentified	Bi	Stems EtOH 80% extract	Suoi Hai, Hanoi	> 200	> 200	> 200	n.d.	n.d.
21	VN-50	*Morinda umbellata* L.	Mặt quỷ	Leaves and branches EtOH 80% extract	Suoi Hai, Hanoi	> 200	> 200	> 200	n.d.	n.d.
22 23	VN-51	*Zanthoxylum nitidum* (Roxb.) DC.	Náng nhà	Stems and twigs EtOH 80% extract	Suoi Hai, Hanoi	30 ± 4	46 ± 5	140 ± 7	>300	>300

24			Náng nhà	Stems and twigs EtOAc fraction	Suoi Hai, Hanoi	>100	33.5± 8	100	n.d.	n.d.
			Náng nhà	Stems and twigs Aqueous fraction	Suoi Hai, Hanoi	> 200	> 200	> 200	n.d.	n.d.
25	VN-52	Unidentified	Đan vàng	Stems EtOH 80% extract	Suoi Hai, Hanoi	>300	>300	>300	>300	>300
26	VN-53	Unidentified	Cây cháy nhà	Stems EtOH 80% extract	Suoi Hai, Hanoi	> 200	> 200	> 200	n.d.	n.d.
25	VN-54	*Achyranthes aspera* L.	Kèng pẹ	Stems EtOH 80% extract	Suoi Hai, Hanoi	>300	>300	>300	>300	>300
26	VN-55	*Rourea minor* (Gaertn.) Merr.	Ngồng chan	Stems EtOH 80% extract	Suoi Hai, Hanoi	> 200	> 200	> 200	n.d.	n.d.
27	VN-56	*Trevesia palmata* (Roxb. ex Lindl.) Vis.	Đu đủ rừng	Stems EtOH 80% extract	Suoi Hai, Hanoi	> 200	> 200	> 200	n.d.	n.d.
28	VN-57	*Garcinia xanthochymus* Hook.f.ex T.Anderson	Lá nụ	Leaves and branches EtOH 80% extract	Suoi Hai, Hanoi	130 ± 10	156 ± 12	127 ± 11	>300	>300
29	VN-58	*Ardisia silvestris* Pit.	Địa màng sảy	Leaves and branches EtOH 80% extract	Suoi Hai, Hanoi	> 200	> 200	> 200	n.d.	n.d.
30	VN-59	Unidentified	Nhải liết	Leaves and branches EtOH 80% extract	Suoi Hai, Hanoi	> 200	> 200	> 200	n.d.	n.d.
31	VN-65	*Paramignya trimera* (Oliv.) Burkill	Xáo tam phân	Roots MeOH 100% fraction DIAION	Khanh Hoa Province	42 ± 3	43 ± 4	42 ± 3	84 ± 7	85 ± 6

pharmaceuticals

MDPI

Table S2. TList of primers used for real-time PCR.

Target	Forward 5' – 3'	Reverse 5' – 3'
p53	TGTGACTTGCACGTACTCCC	ACCATCGCTATCTGAGCAGC
MDM2	TCTGTGAGTGAGAACAGGTGTC	TGGCGTTTTCTTTGTCGTTCA
Puma	ATGAGCCAAACCTGACCACT	TGAGATGGATGGGGATTGGG
Bax	GACTCCCCCCGAGAGGTCTT	ACAGGGCCTTGAGCACCAGTT
p21	CCGCCCCCTCCTCTAGCTGT	CCCCCATCATATACCCCTAACACA
