# Supplementary material for: Discovery of Benzophenanthridine Alkaloids from Zanthoxylum nitidum That Target the MDM2–p53 Axis in NSCLC
Source: Pharmaceuticals (Basel). 2026 May 22;19(6):814. doi: 10.3390/ph19060814 (PMC13304904; doi:10.3390/ph19060814)
Supplement: Supplementary file 1 [file pharmaceuticals-19-00814-s001.zip › figures.pdf]

pharmaceuticals

MDPI

Figure S1. The impact of MDM2 knockdown on the protein levels of p53 and Snail in A549 cell line. The diagrams show the results of quantification performed using the Image J software. Student's *t*-test was used to determine pairwise statistically significant differences. Asterisks represent a significant difference in protein expression (*** $p \leq 0.001$).

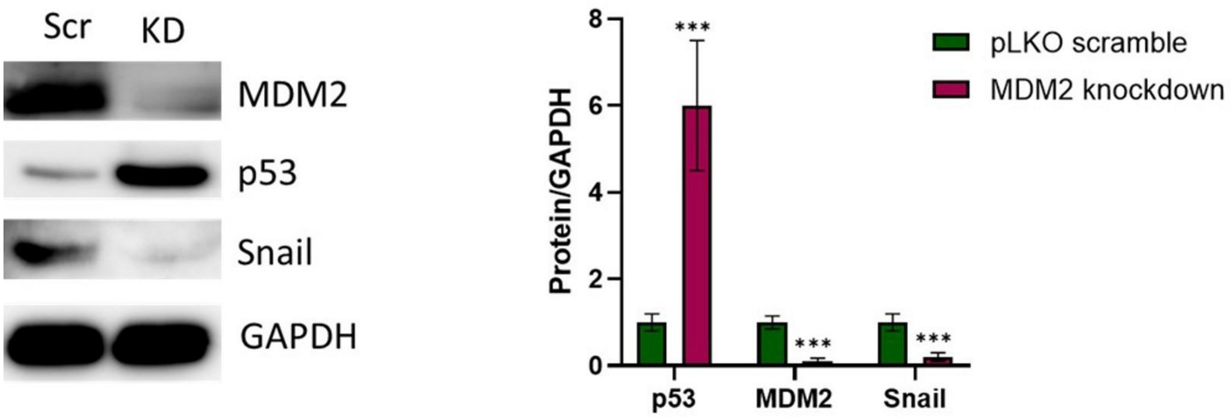

Figure S2. ^{1}H-NMR spectrum of 8-acetonyldihydrochelerythrine (**1**)

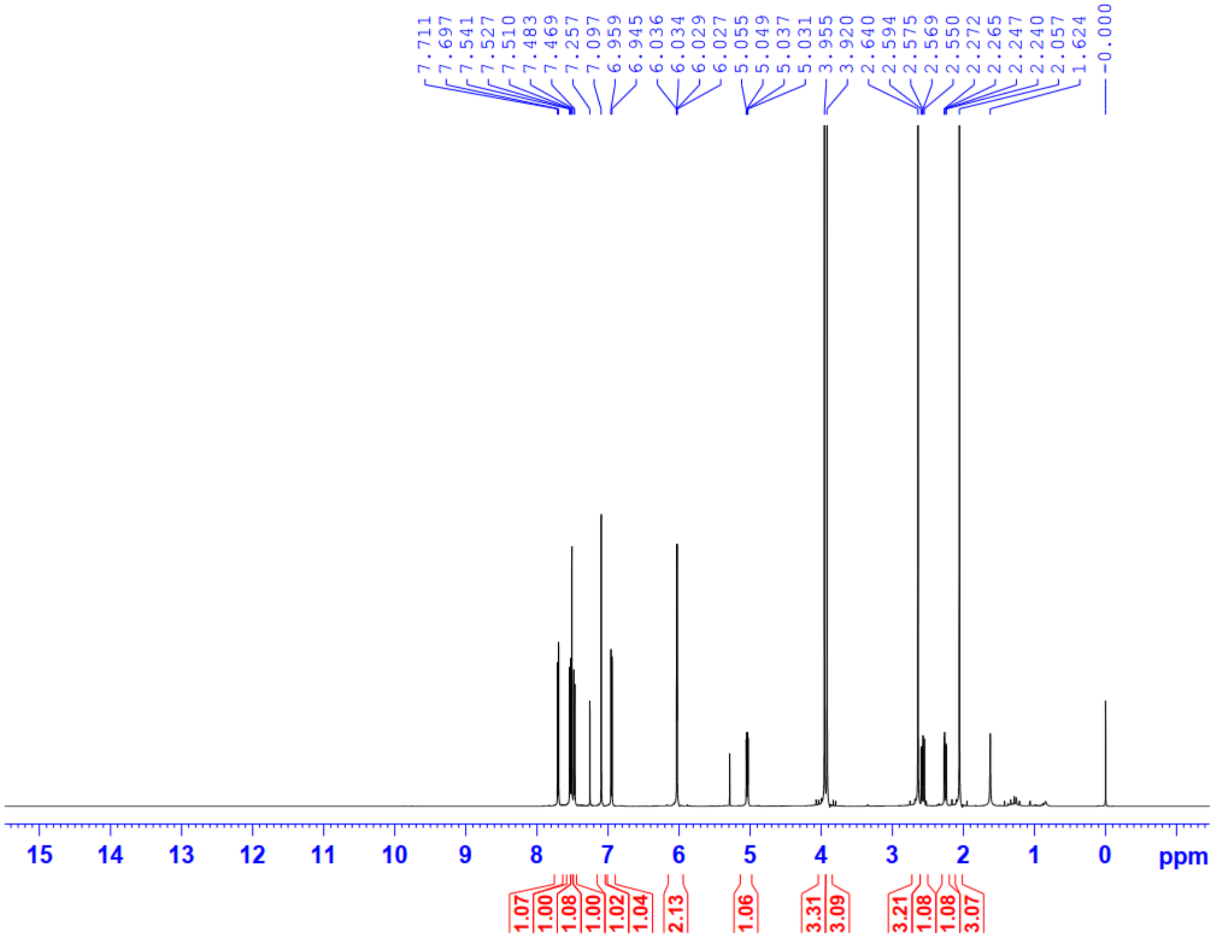

7.711
7.697
7.541
7.527
7.510
7.483
7.469
7.257
7.097
6.959
6.945
6.036
6.034
6.029
6.027
5.055
5.049
5.037
5.031
3.955
3.920
2.640
2.594
2.575
2.569
2.550
2.272
2.265
2.247
2.240
2.057
1.624
0.000
15 14 13 12 11 10 9 8 7 6 5 4 3 2 1 0 ppm
1.07
1.00
1.08
1.00
1.02
1.04
2.13
1.06
3.31
3.09
3.21
1.08
1.08
3.07

Figure S3. ^{13}C-NMR spectrum of 8-acetonyldihydrochelerythrine (**1**)

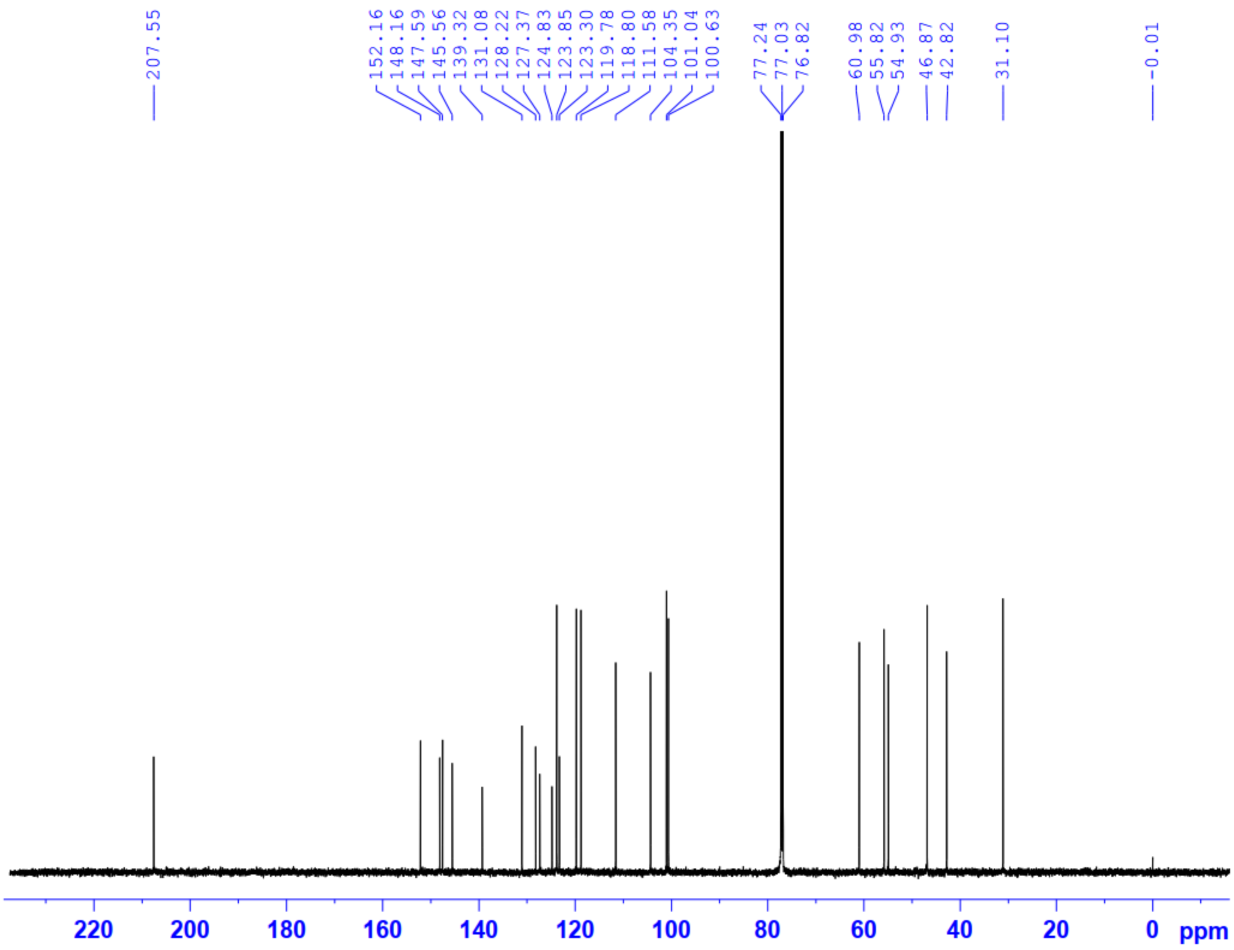

Figure S4. DEPT spectrum of 8-acetonyldihydrochelerythrine (**1**)

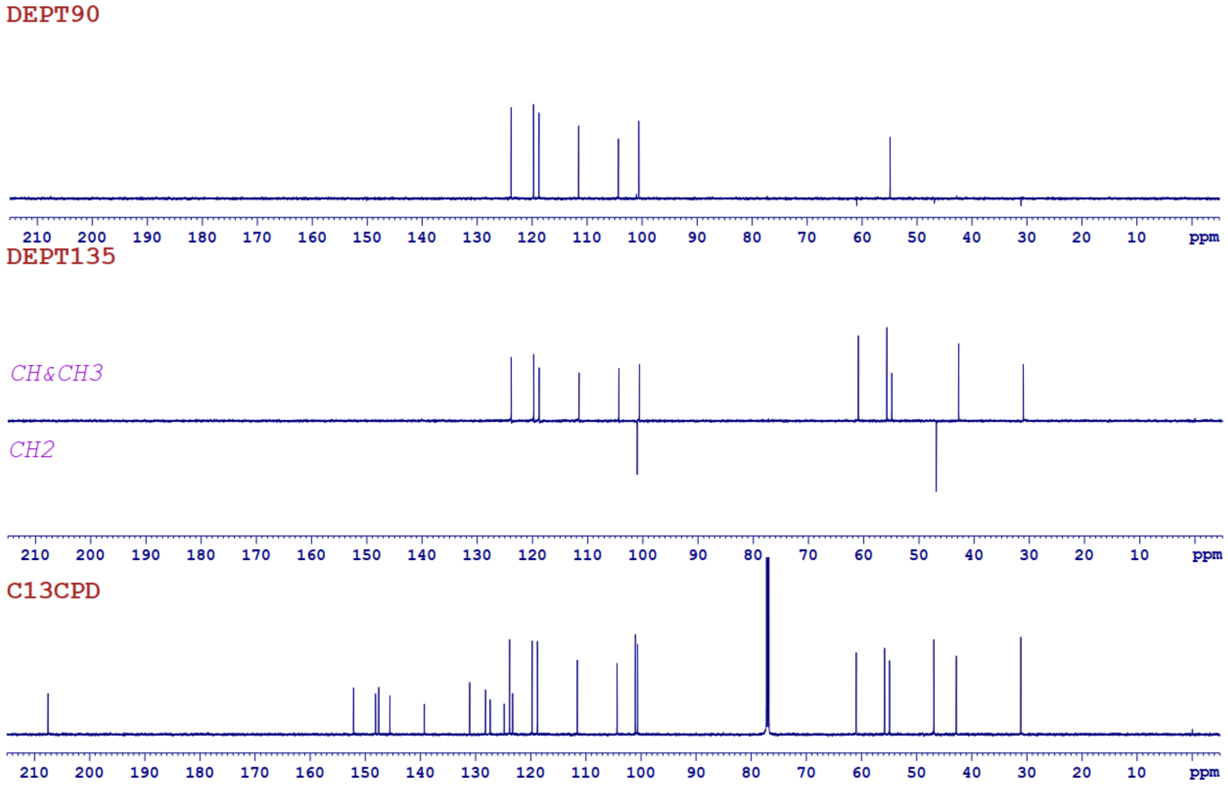

Figure S5. HR-ESI-MS spectrum of 8-acetonyldihydrochelerythrine (**1**)

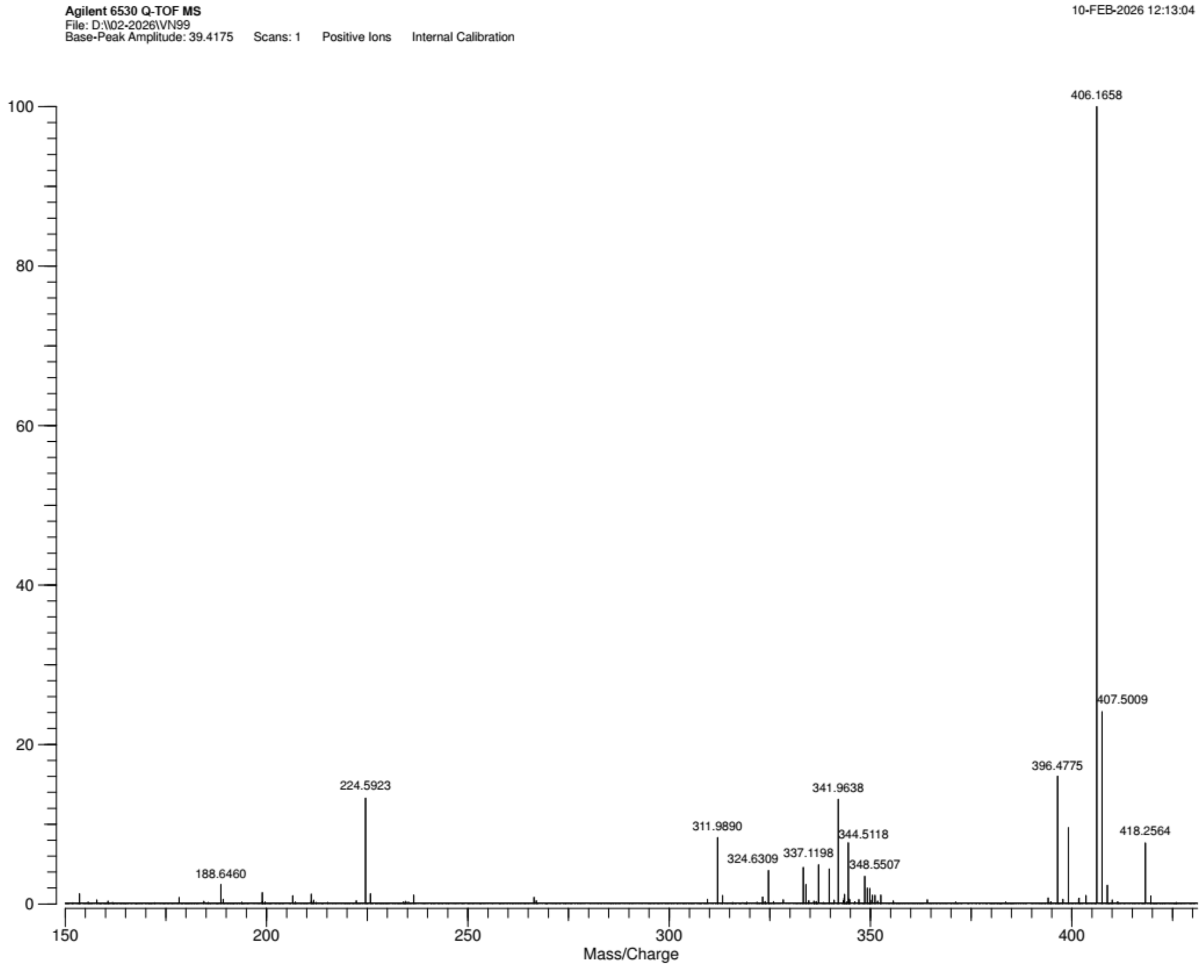

Figure S6. ^{1}H-NMR spectrum of nitidine (**2**)

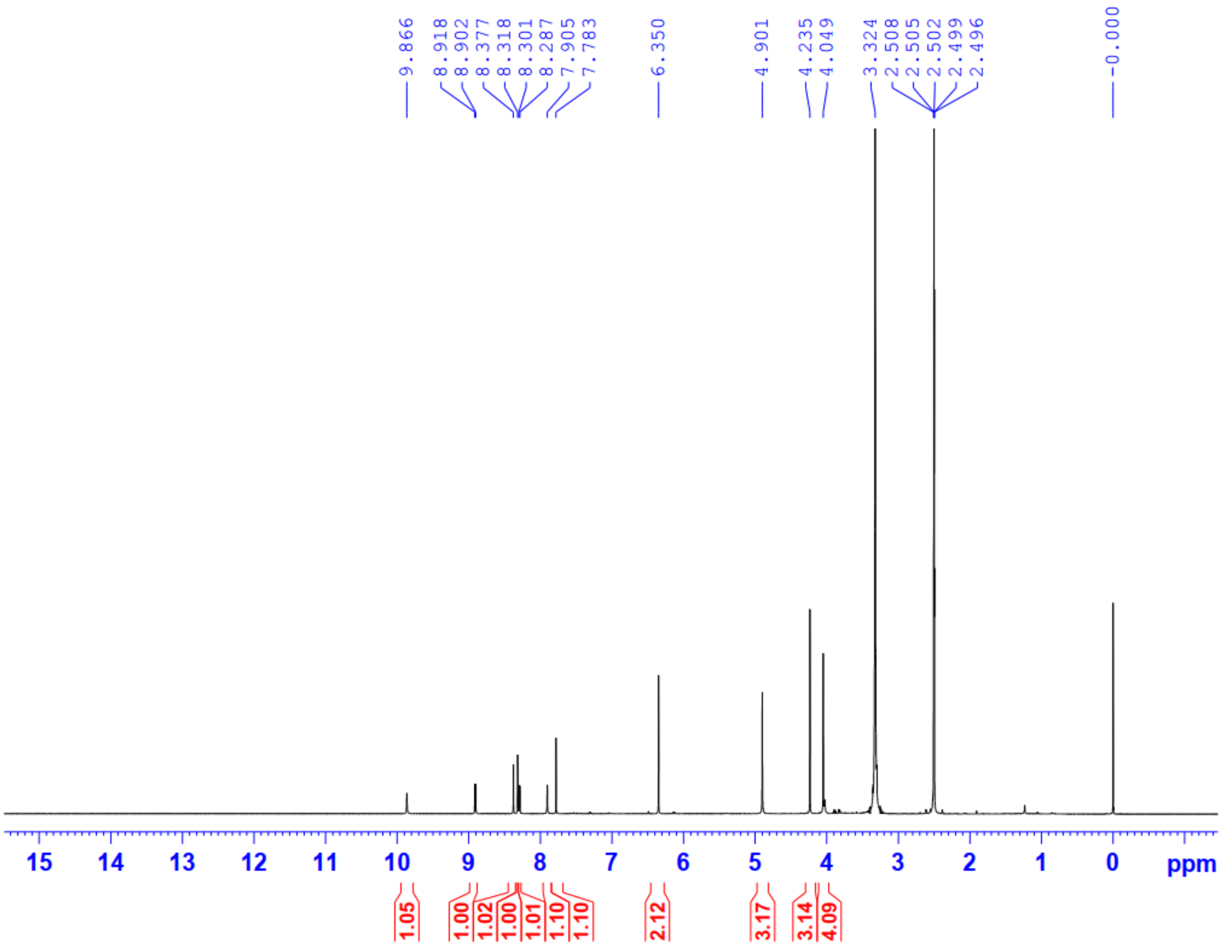

Figure S7. ^{13}C-NMR spectrum of nitidine (**2**)

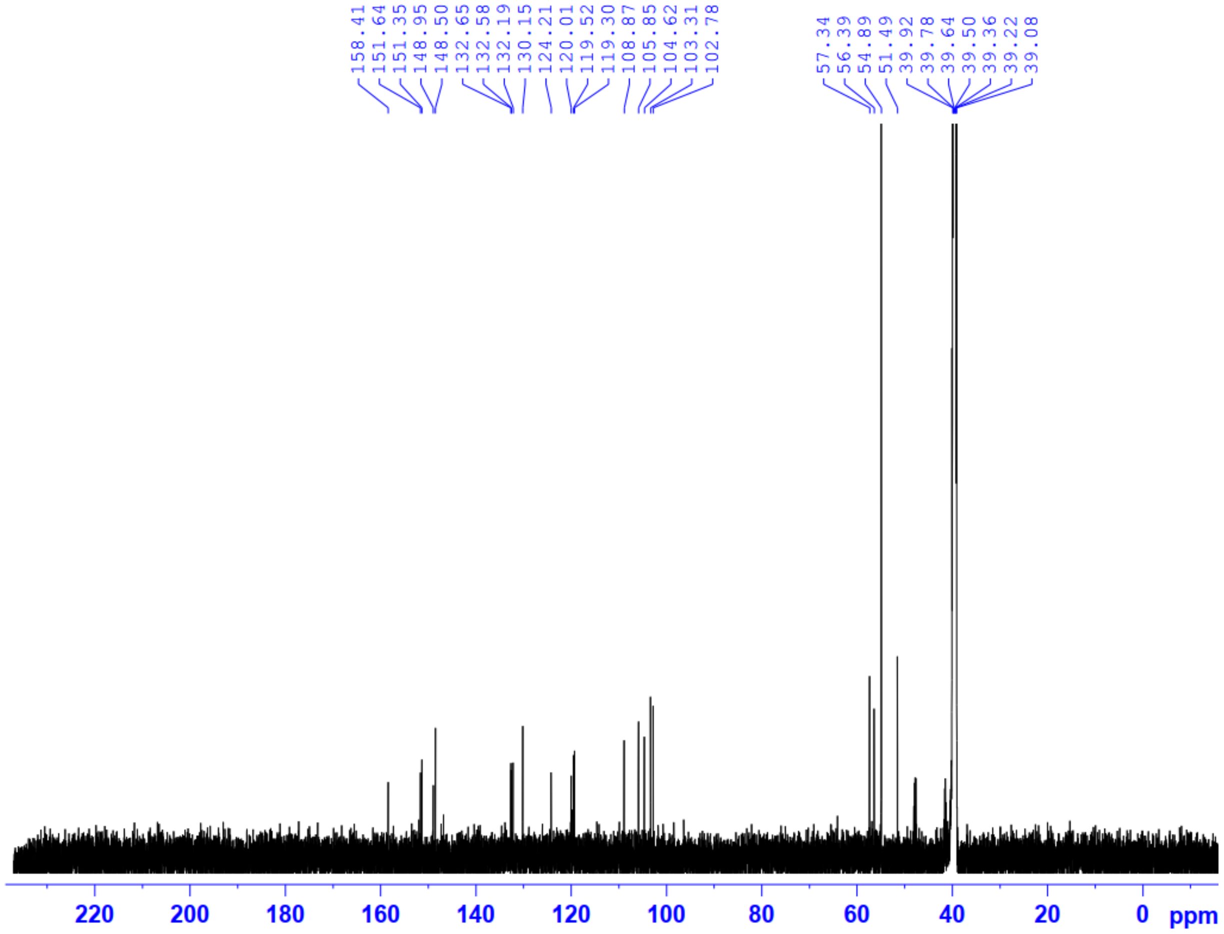

Figure S8. HR-ESI-MS spectrum of nitidine (**2**)

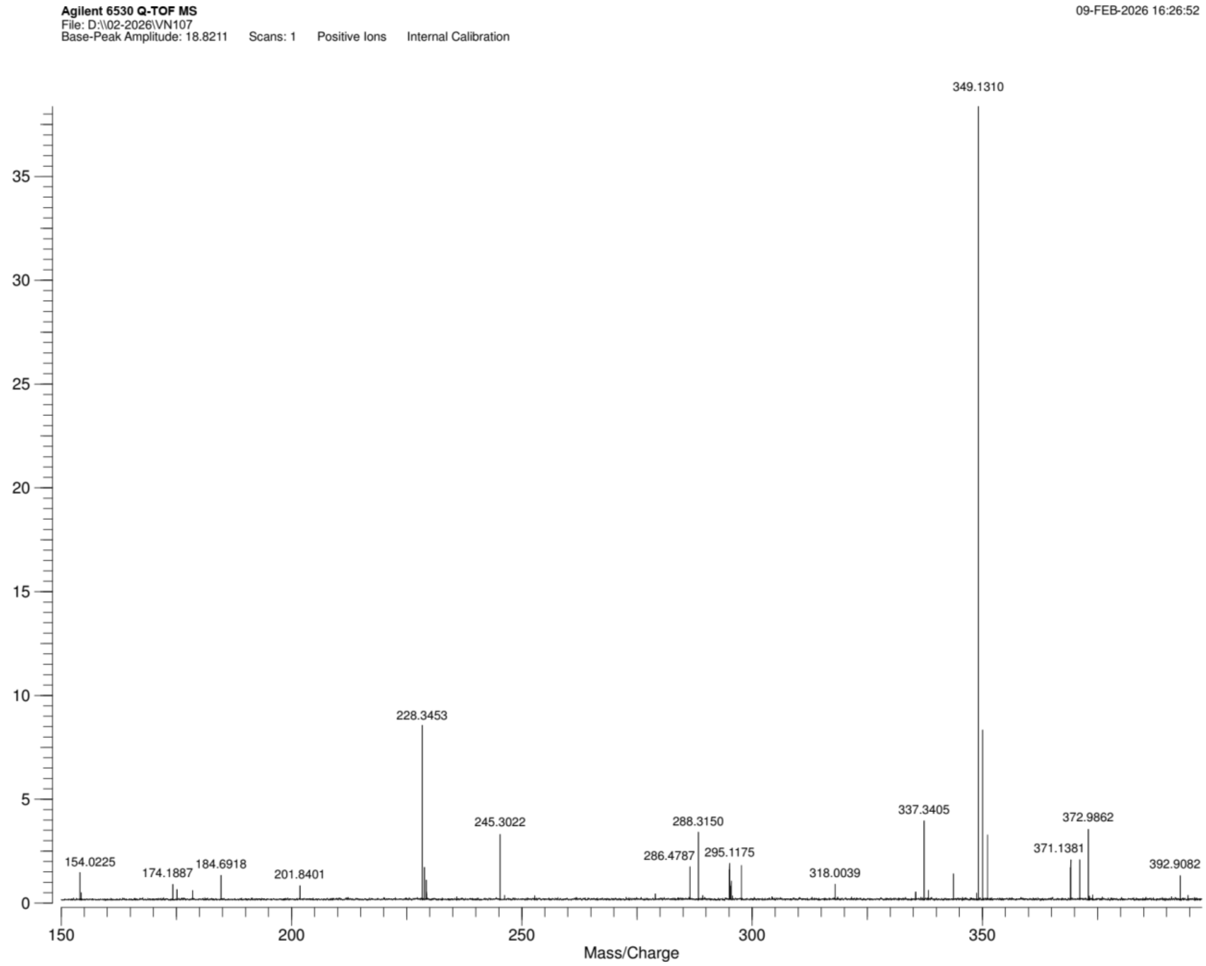

Figure S9. ^{1}H-NMR spectrum of terihanine (**3**)

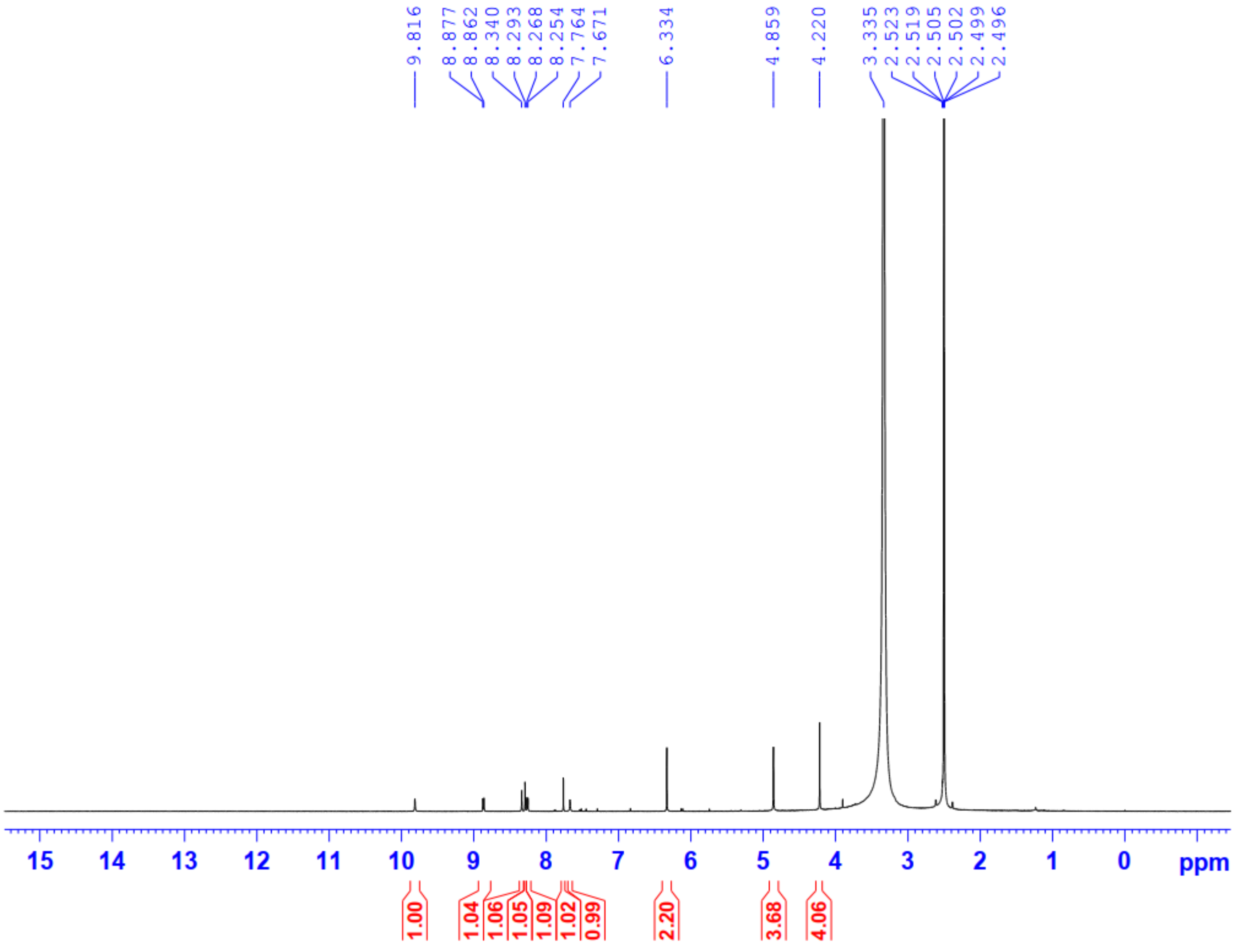

Figure S10. HR-ESI-MS spectrum of terihanine (**3**)

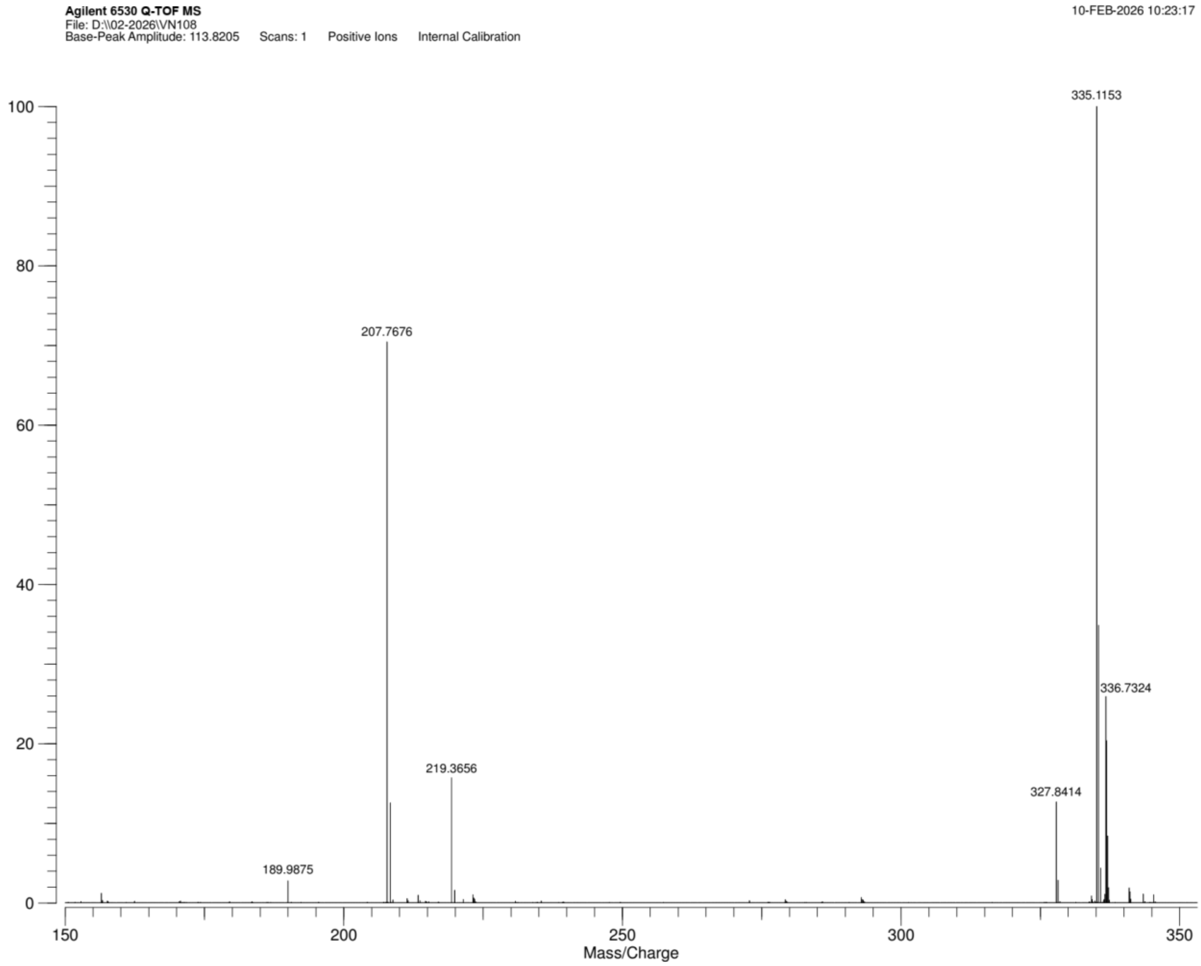

Figure S11. ^{13}C-NMR spectrum of terihanine (**3**)

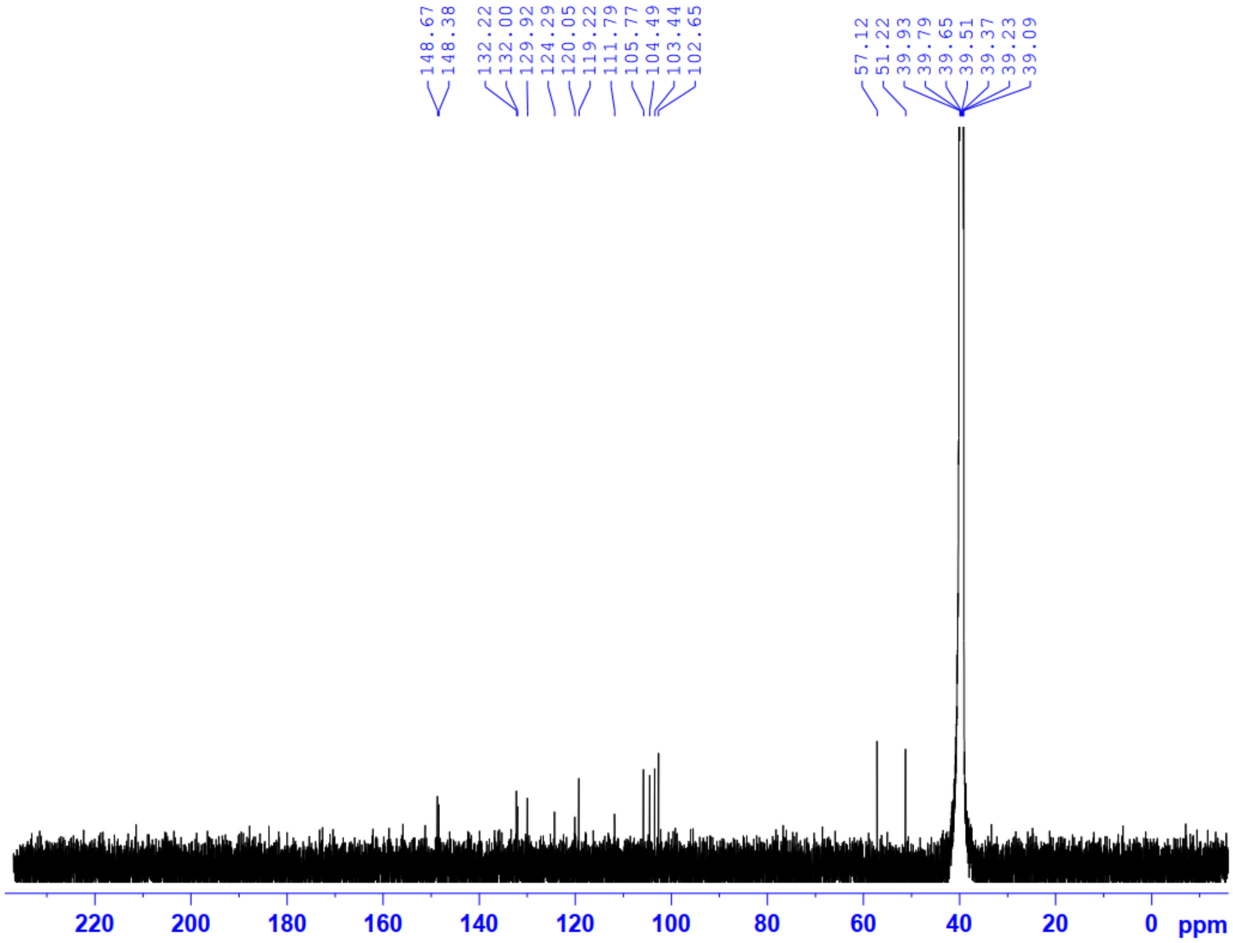

Figure S12. ^{1}H-NMR spectrum of sanguinarine (**4**)

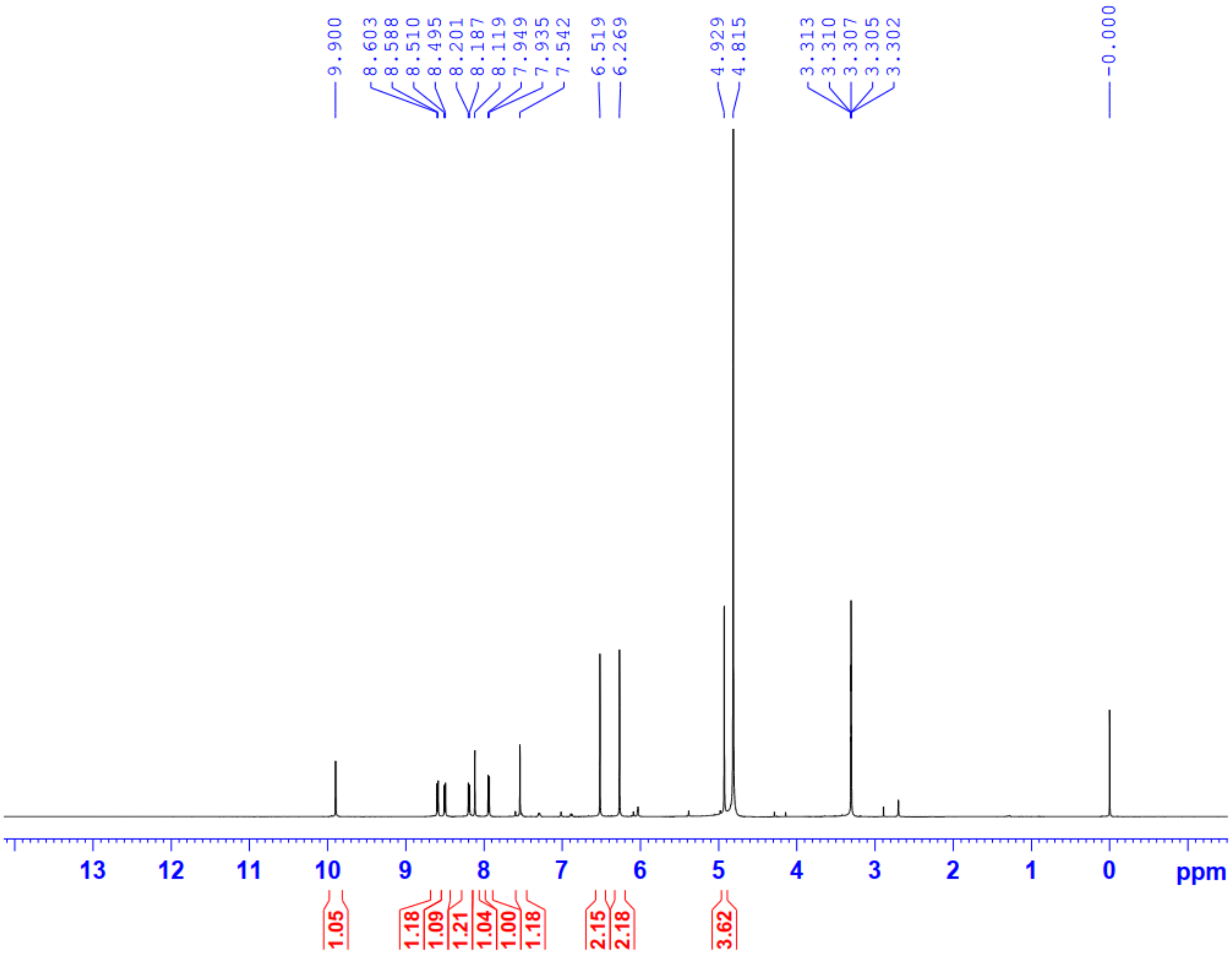

Figure S13. ^{13}C-NMR spectrum of sanguinarine (**4**)

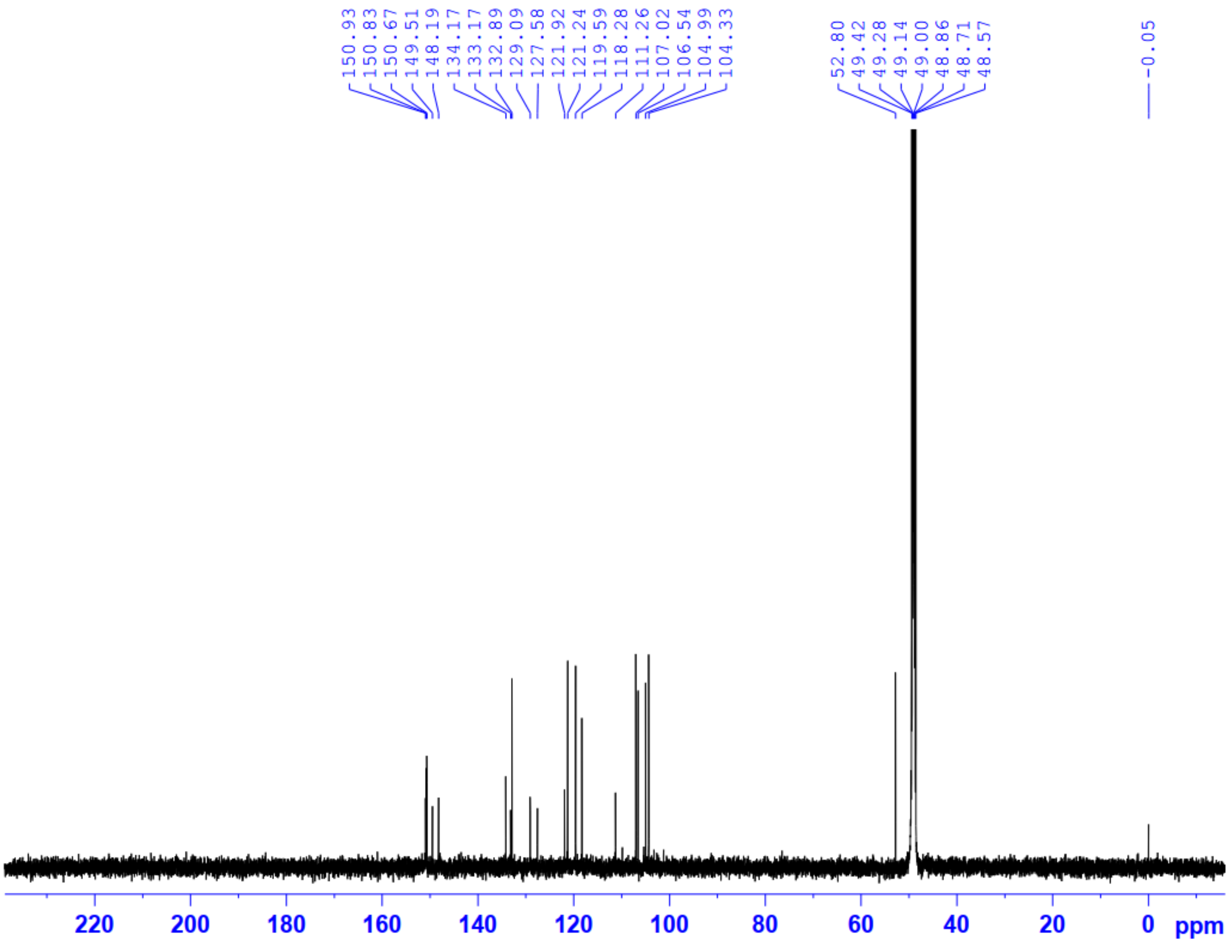

Figure S14. DEPT spectrum of sanguinarine (**4**)

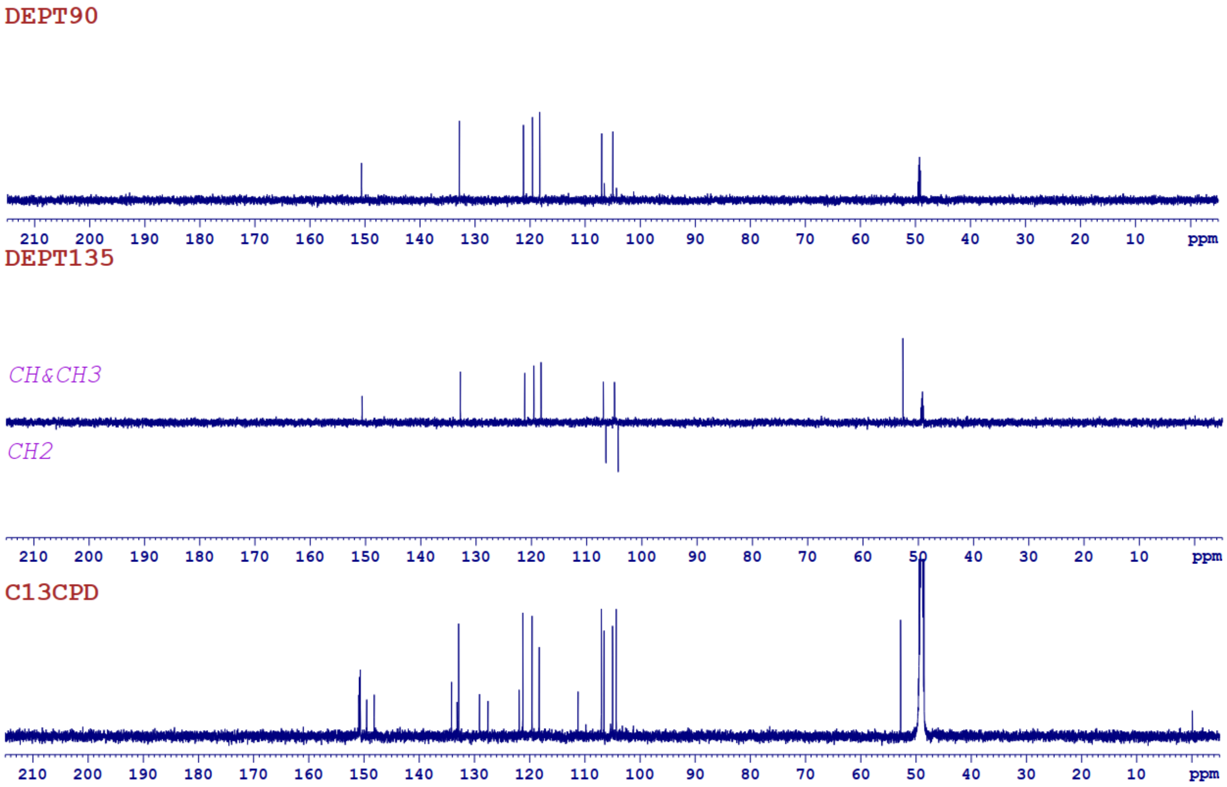

Figure S15. HR-ESI-MS spectrum of sanguinarine (**4**)

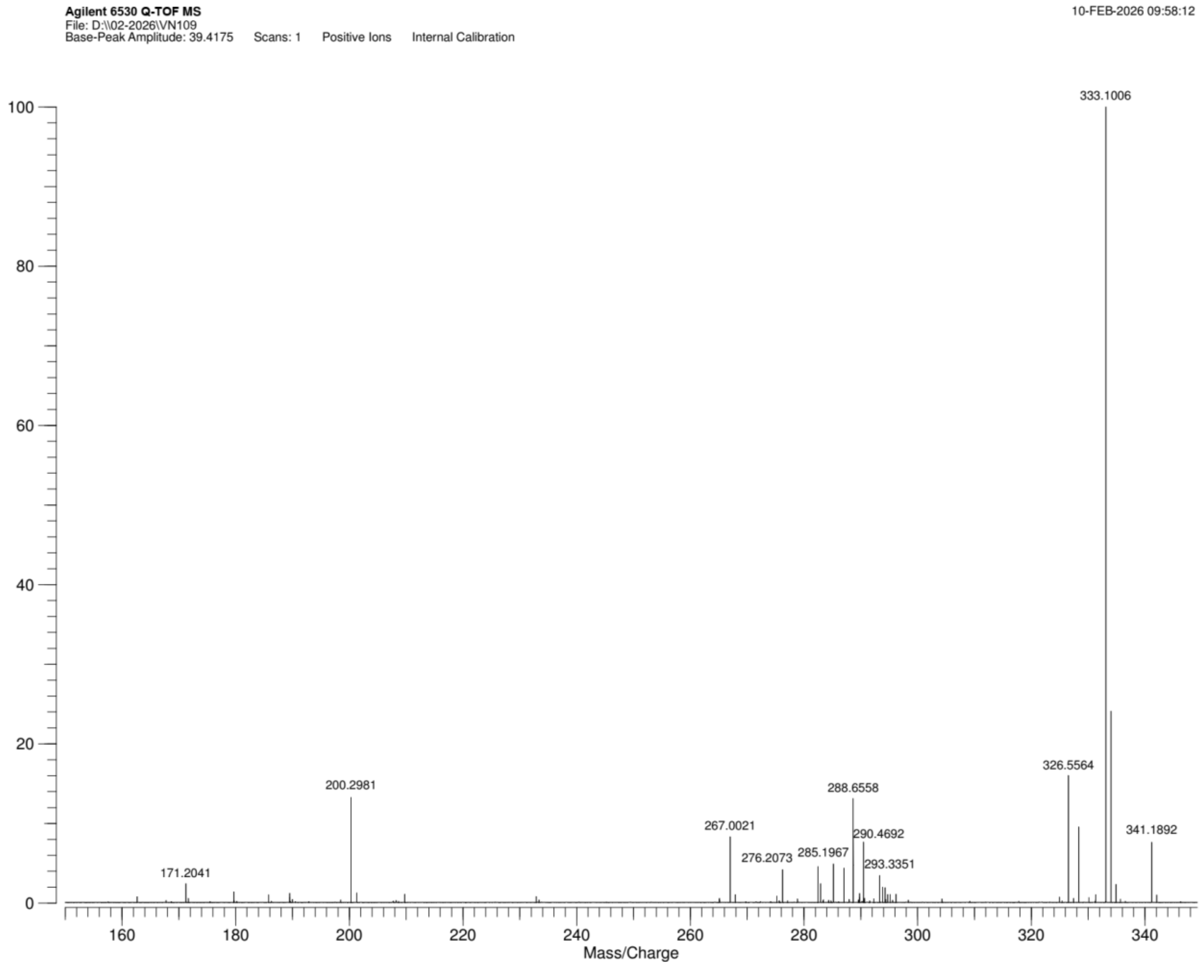

Figure S16. ^{1}H-NMR spectrum of sesamin (**5**)

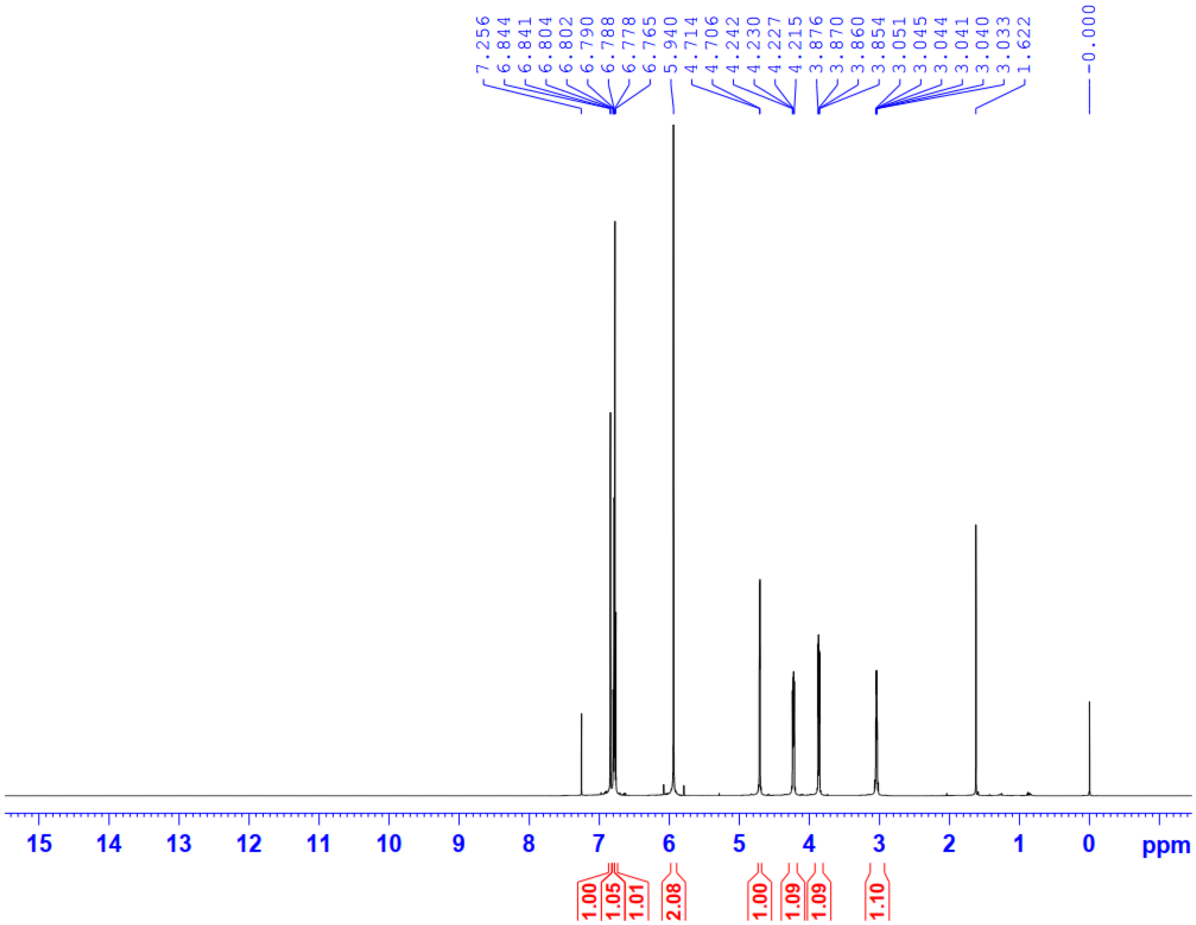

Figure S17. HR-ESI-MS spectrum of sesamin (**5**)

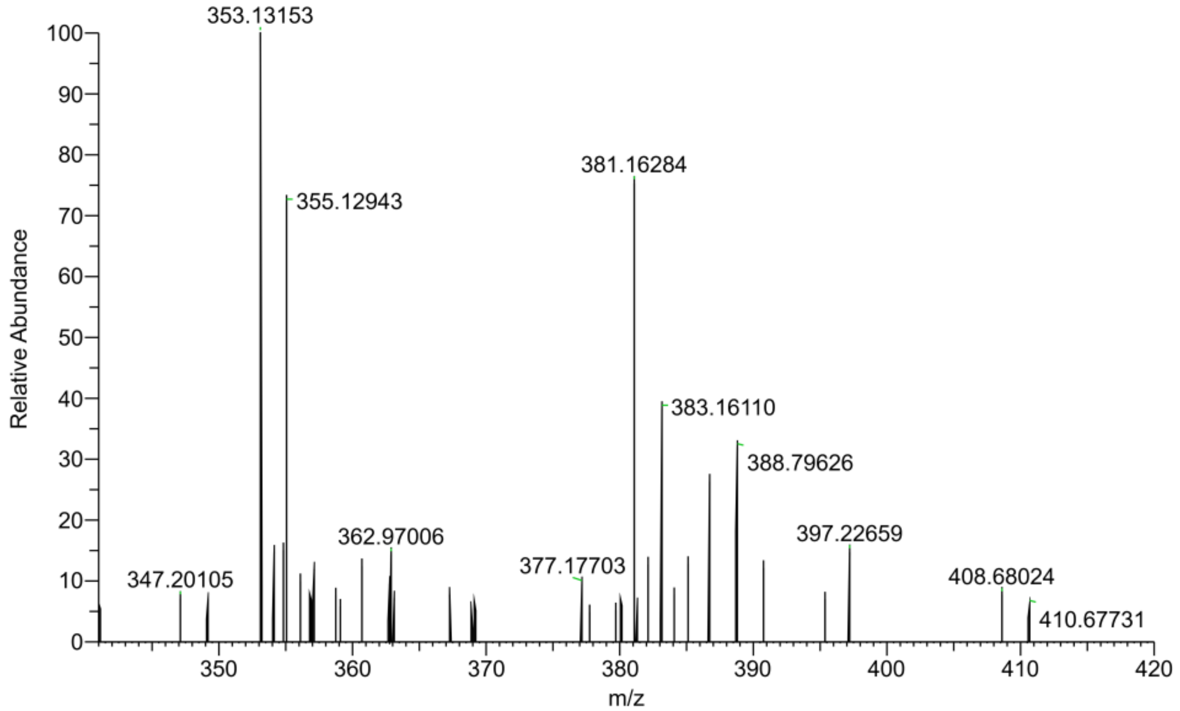

Figure S18. ^{1}H-NMR spectrum of myo-inositol (**6**)

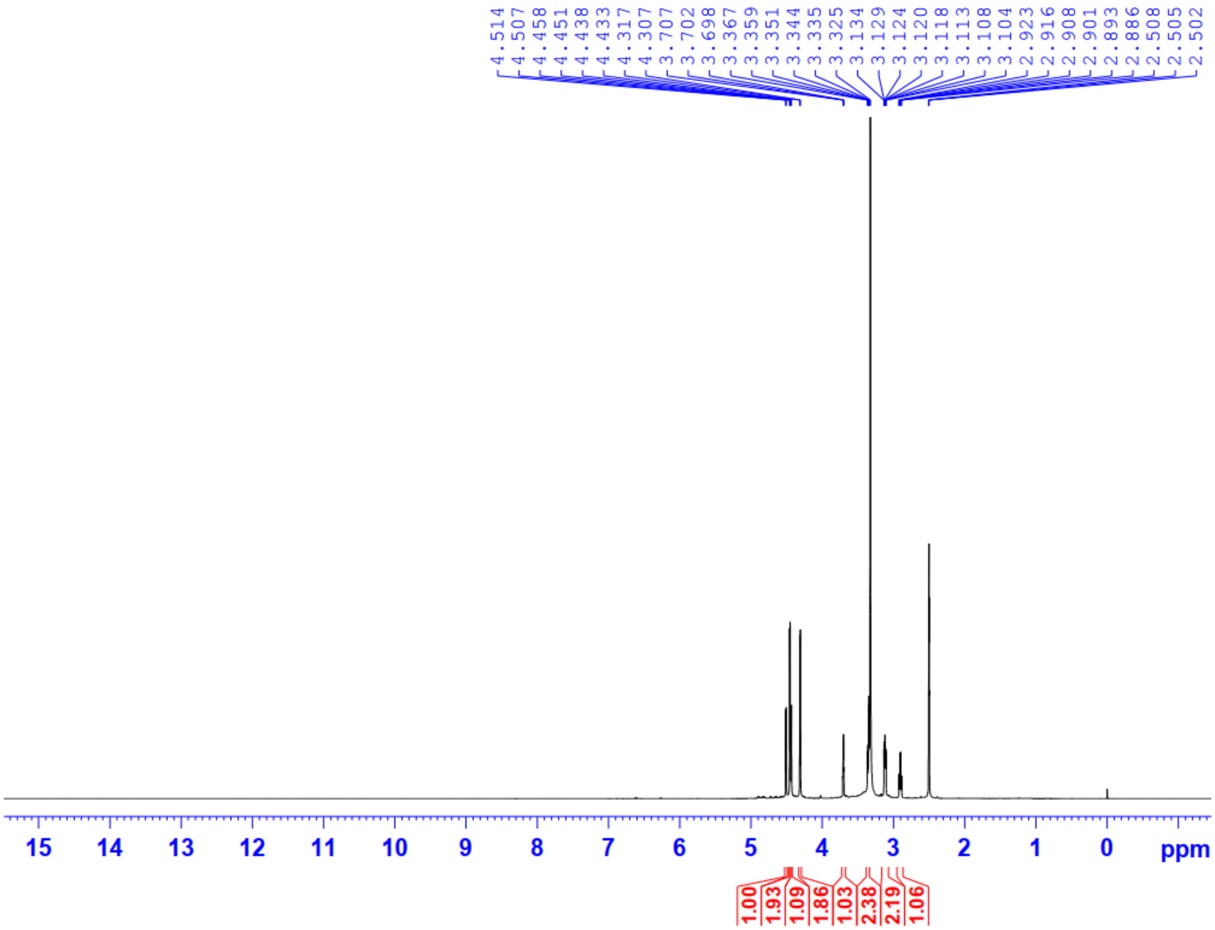

Figure S19. HR-ESI-MS spectrum of myo-inositol (**6**)

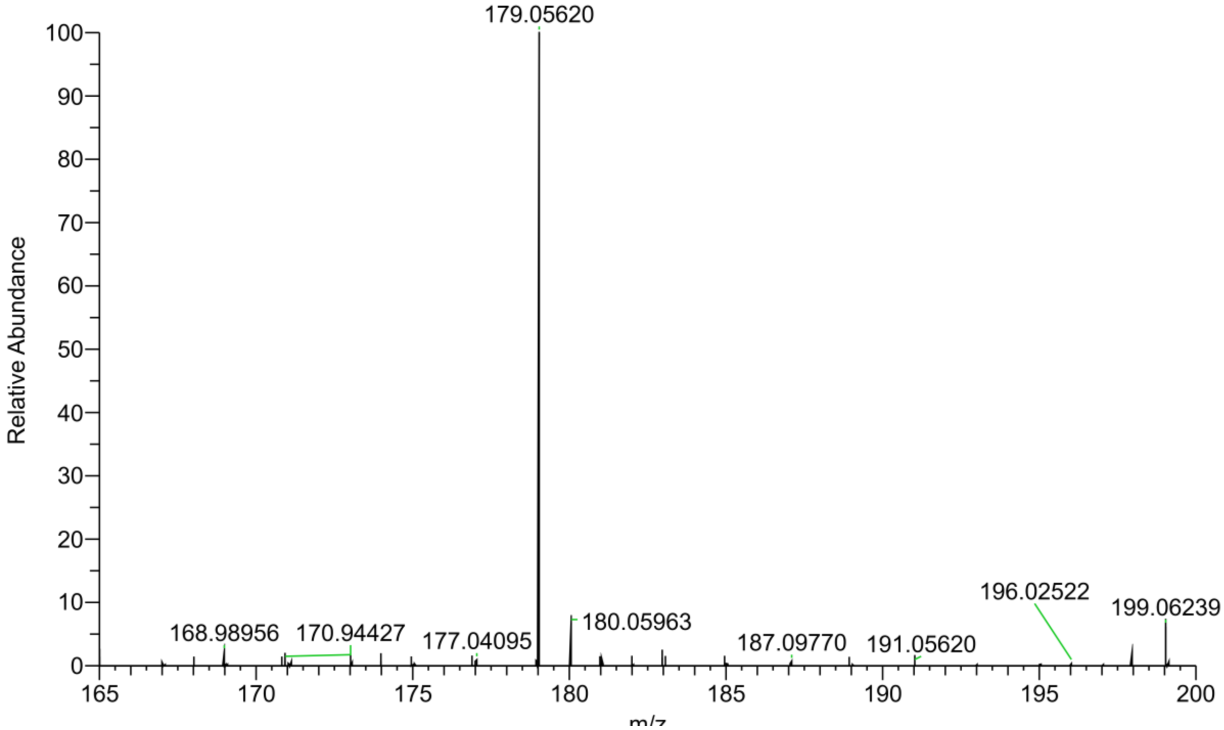

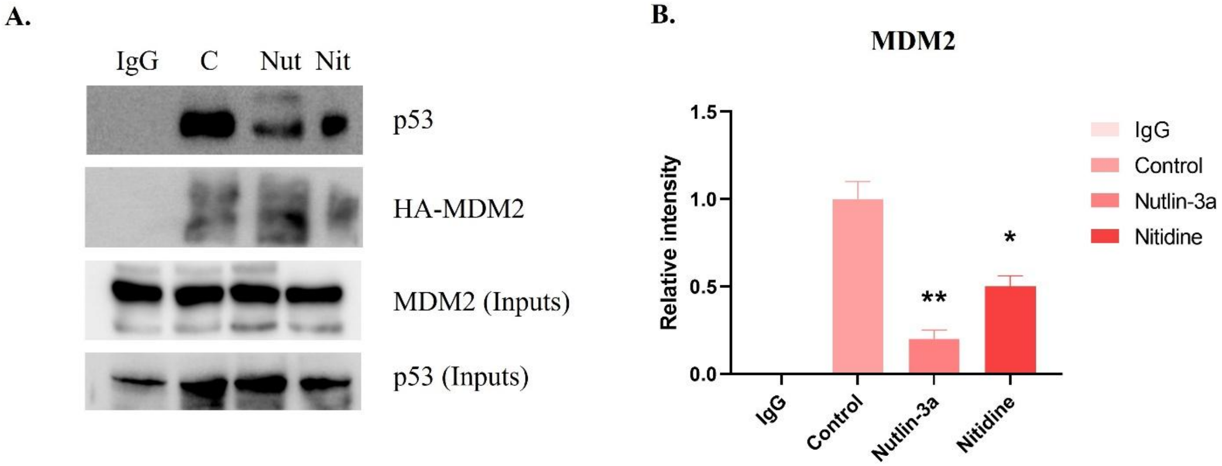

Figure S20. Nitidine interferes with MDM2-p53 binding. Western-blot demonstrates the results of co-immunoprecipitation of HA-tagged MDM2 with endogenous p53 in DMSO- (control) and nitidine-treated A549 cells. Nutlin-3a was used as a positive control. Nit—nitidine, Nut—nutlin-3a, IgG—control antibody. The diagrams show the results of quantification performed using the ImageJ software. Student's *t*-test was used to determine pairwise statistically significant differences. Asterisks represent a significant difference in protein expression (* $p \leq 0.05$; ** $p \leq 0.01$).

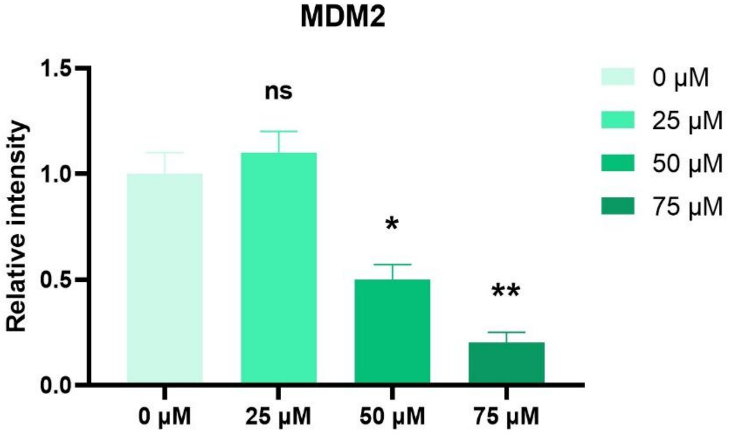

Figure S21. Nitidine affects the protein levels of p53, MDM2, Snail, and β-catenin in H1975 cell line bearing mutant p53. Western-blot. The diagrams show the results of quantification performed using the Image J software. Student's *t*-test was used to determine pairwise statistically significant differences. Asterisks represent a significant difference in protein expression (* $p \leq 0.05$; ** $p \leq 0.01$).

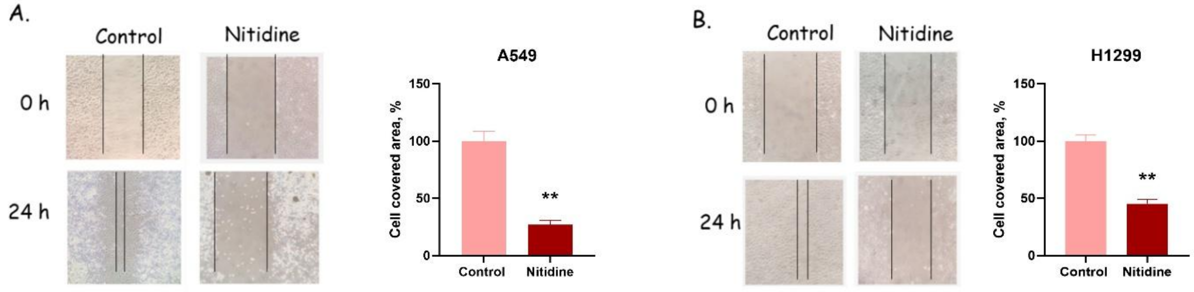

Figure S22. The impact of nitidine treatment (40 μM, 24 h) on wound closure in A549 and H1299 cell lines. Wound-healing assay. In parallel experiment, trypan blue staining was used for the assessment of viability. Finally, the rate of wound closure was normalized to the number of viable cells. A Student's *t*-test was used to determine statistically significant pairwise differences. Asterisks represent significant differences (* $p \leq 0.05$; ** $p \leq 0.01$).
